# Regularized Bayesian transfer learning for population-level etiological distributions

**DOI:** 10.1093/biostatistics/kxaa001

**Published:** 2020-02-10

**Authors:** Abhirup Datta, Jacob Fiksel, Agbessi Amouzou, Scott L Zeger

**Affiliations:** 1 Department of Biostatistics, Johns Hopkins University, 615 North Wolfe Street, Baltimore, MD 21205, USA; 2 Department of International Health, Johns Hopkins University, 615 North Wolfe Street, Baltimore, MD 21205, USA

**Keywords:** Bayesian, Classification, Epidemiology, Hierarchical modeling, Regularization, Transfer learning, Verbal autopsy

## Abstract

Computer-coded verbal autopsy (CCVA) algorithms predict cause of death from high-dimensional family questionnaire data (*verbal autopsy*) of a deceased individual, which are then aggregated to generate national and regional estimates of cause-specific mortality fractions. These estimates may be inaccurate if CCVA is trained on non-local training data different from the local population of interest. This problem is a special case of *transfer learning*, i.e., improving classification within a target *domain* (e.g., a particular population) with the classifier trained in a *source-domain*. Most transfer learning approaches concern individual-level (e.g., a person’s) classification. Social and health scientists such as epidemiologists are often more interested with understanding etiological distributions at the population-level. The sample sizes of their data sets are typically orders of magnitude smaller than those used for common transfer learning applications like image classification, document identification, etc. We present a parsimonious hierarchical Bayesian *transfer learning* framework to directly estimate population-level class probabilities in a target domain, using any baseline classifier trained on source-domain, and a small labeled target-domain dataset. To address small sample sizes, we introduce a novel shrinkage prior for the transfer error rates guaranteeing that, in absence of any labeled target-domain data or when the baseline classifier is perfectly accurate, our transfer learning agrees with direct aggregation of predictions from the baseline classifier, thereby subsuming the default practice as a special case. We then extend our approach to use an ensemble of baseline classifiers producing an unified estimate. Theoretical and empirical results demonstrate how the ensemble model favors the most accurate baseline classifier. We present data analyses demonstrating the utility of our approach.

## 1. Introduction

*Verbal autopsy*—A survey of the household members of a deceased individual, act as a surrogate for medical autopsy report in many countries. Computer-coded verbal autopsy (CCVA) algorithms are high-dimensional classifiers that predict cause of death (COD) from these high-dimensional family questionnaires which are then aggregated to generate national and regional estimates of cause-specific mortality fractions (CSMF). These estimates may be inaccurate as CCVA are usually trained using non-local information not representative of the local population of interest. This problem is a special case of *transfer learning*, a burgeoning area in statistics and machine learning.

Classifiers trained on *source-domain* data tend to predict inaccurately in a *target domain* different from the source-domain in terms of marginal and conditional distributions of the *features* (covariates) and *labels* (responses) (Shimodaira, 2000). Various *domain adaptation* strategies have been explored for transfer learning of generic classifiers which adjust for this distributional difference between the two domains. We refer the readers to Weiss *and others* (2016) and Pan and Yang (2010) for a comprehensive review of transfer learning for classification problems. We focus on the setting where there is abundant labeled source-domain data, abundant unlabeled target-domain data, and limited labeled target data. Transfer learning approaches pertaining to this setting include multi-source-domain adaptation (CP-MDA, Chattopadhyay *and others*, 2012), neural networks (TCNN, Oquab *and others*, 2014), adaptive boosting (TrAdaBoost, Dai *and others*, 2007; Yao and Doretto, 2010), feature augmentation method (FAM, Daumé III, 2009), spectral feature alignment (SFA, Pan *and others*, 2010) among others.

All of the aforementioned transfer learning classification approaches are motivated by applications in image, video or document classification, text sentiment identification, and natural language processing where individual classification is the goal. Hence, they usually focus on the individual’s (e.g., a person’s or an image’s) classification within a target domain (e.g., a particular population) with training performed in data from a different source-domain.

Social and health scientists such as epidemiologists are often more interested with understanding etiological distributions at the population-level rather than classifying individuals. For example, we aim to estimate national and regional estimates of cause-specific fractions of child mortality. Hence, our goal is not individual prediction but rather transfer learning of population-level class probabilities in the target domain. None of the current transfer learning approaches are designed to directly estimate population-level class membership probabilities.

Additionally, the extant transfer learning approaches rely on large source-domain databases of millions of observations for training the richly parameterized algorithms. The sample sizes of datasets in epidemiology are typically orders of magnitude smaller. Most epidemiological applications use field data from surveys, leading to databases with much smaller sample sizes and yet with high-dimensional covariates (survey records). For example, in our application, the covariate space is high-dimensional ($\sim$200–350 covariates), the “abundant” source-domain data have around $\sim$2000 samples, while the local labeled data can have as few as 20–100 samples. Clearly, in such cases, the local labeled data are too small to train a classifier on a high-dimensional set of covariates, as the resulting estimates will be highly variable. A baseline classifier trained on the larger source-domain data will tend to produce more stable estimates, but the high precision will come at the cost of sacrificing accuracy if the source- and target-domains differ substantially.

Our parsimonious solution to this bias-variance trade-off problem is to use the baseline classifier trained on source-domain information to obtain an initial prediction of target-domain class probabilities, but then refine it with the labeled target-domain data. We proffer a hierarchical Bayesian framework that unifies these two steps. With $C$ classes and $S$-dimensional covariates, the advantage of this new approach is that the small labeled data for the target domain is only used to estimate the $C \times C$*confusion matrix* of the *transfer error* (misclassification) rates instead of trying to estimate $\mathcal O(SC)$ parameters of the classifier directly from the target-domain data. Since $S\gg C$, this approach considerably reduces the dimensionality of the problem. To ensure a stable estimation of the confusion matrix, we additionally use a regularization prior that shrinks the matrix towards identity unless there is substantial transfer error. We show that, in the absence of any target-domain labeled data or in case of zero transfer error, posterior means of class probability estimates from our approach coincide with those from the baseline learner, establishing that the naive estimation that ignores transfer error is a special case of our algorithm. We devise a novel, fast Gibbs sampler with augmented data for our Bayesian hierarchical model.

We then extend our approach to one that uses an ensemble of input predictions from multiple classifiers. The ensemble model accomplishes method-averaging over different classifiers to reduce the risk of using one method that is inferior to others in a particular study. We establish a theoretical result that the class probability estimates from the ensemble model coincides with that from a classifier with zero transfer error. A Gibbs sampler for the ensemble model is also developed, as well as a computationally lighter version of the model that is much faster and involves fewer parameters. Simulation and data analyses demonstrate how the ensemble sampler consistently produces estimates similar to those produced by using our transfer learning on the single best classifier.

Our approach is also *post hoc*, i.e., only uses pre-trained baseline classifier(s), instead of attempting to retrain the classifier(s) multiple times with different versions of training data. This enables us to use publicly available implementations of these classifier(s) and circumvents iterative training runs of the baseline classifier(s) which can be time-consuming and inconvenient in epidemiological settings where data collection continues for many years, and the class probabilities needs to be updated continually with the addition of every new survey record. The *post hoc* approach also ensures we can work with non-statistical classifiers that do not use a training data but some sort of source-domain information (e.g., CCVA algorithms InterVA and EAVA).

The rest of the manuscript is organized as follows. We present the motivating application in Section 1.1. In Sections 2 and 3, we present the methodology and its extension to the ensemble case. Section 4 considers the extension where class probabilities can be modeled as a function of a few covariates like age, sex, seasons, spatial regions, etc. Section 5 presents simulation results. Section 6 returns to the motivating dataset and uses our transfer learning model to estimate national CSMFs for children deaths in India and Tanzania. We end the manuscript in Section 7 with a discussion of limitations and future research opportunities. A glossary of the different abbreviations used throughout the text is. provided in Table 1.

**Table 1.** Glossary of acronyms used in the manuscript

Acronym	Full form	Acronym	Full form
VA	Verbal autopsy	PHMRC	Population Health Metrics Research Consortium
CCVA	Computer-coded VA	COMSA	Countrywide Mortality Surveillance for Action
COD	Cause of Death	CSMF	Cause-Specific Mortality Fraction
CSMFA	CSMF accuracy	GS-COD	Gold-standard Cause of Death

### 1.1. Motivating dataset

In low- and middle-income countries, it is infeasible to conduct full autopsies for the majority of deaths due to economic and infrastructural constraints, and/or religious or cultural prohibitions against autopsies (AbouZahr *and others*, 2015; Allotey *and others*, 2015). An alternative method to infer the COD (or “etiology”) is to conduct *verbal autopsy* (VA)—a systematic interview of the relatives of the deceased individual—to obtain information about symptoms observed prior to death (Soleman *and others*, 2006). Statisticians have developed several specialized classifiers that predict COD using the high-dimensional VA records as input. Examples include Tariff (James *and others*, 2011; Serina *and others*, 2015), InterVA (Byass *and others*, 2012), InSilicoVA (McCormick *and others*, 2016), the King and Lu method (King *and others*, 2008), EAVA or expert algorithm (Kalter *and others*, 2015), etc. Software for many of these algorithms are publicly available, e.g., Tariff (Li *and others*, 2018c), InSilicoVA (Li *and others*, 2018a), InterVA (Thomas *and others*, 2018), and the openVA R-package (Li *and others*, 2018b) has consolidated most of these individual software into a single package. Generic classifiers like random forests (Breiman, 2001), naive Bayes classifiers (Minsky, 1961), and support vector machines (Cortes and Vapnik, 1995) have also been used (Flaxman *and others*, 2011; Miasnikof *and others*, 2015; Koopman *and others*, 2015) for classifying verbal autopsies. Predicted COD labels for each VA record in a nationally representative VA database is aggregated to obtain national CSMF—the population-level class membership probabilities, that are often the main quantities of interest for epidemiologists, local governments, and global health organizations.

Formally, a CCVA algorithm is simply a classifier using the $S \times 1$ covariate vector (VA report) $\mathbf s$ to predict $c$—one of $C$ possible COD categories. Owing to the high dimensionality of the covariate space (VA record consists of responses to 200–350 questions), learning this mapping $P(c \;|\; {\mathbf s})$ requires substantial amount of *gold standard* (labeled) training data. Usually in the country of interest, VA records are available for a large representative subset of the entire population, but gold standard cause of death (GS-COD) is ascertained for only a very small fraction of these deaths. In other words, there is abundant unlabeled data but extremely limited labeled data in the target domain. The ongoing project Countrywide Mortality Surveillance for Action (COMSA) Mozambique typify this circumstance, where, in addition to conducting a nationally representative VA survey, researchers will have access to gold standard COD for a small number of deaths from one or two local hospitals using minimally invasive autopsies (MIA) (Byass, 2016). Budgetary constraints and socio-cultural factors unfortunately imply that only a handful of deaths can eventually be autopsied (up to a few hundred).

**Fig. 1:**
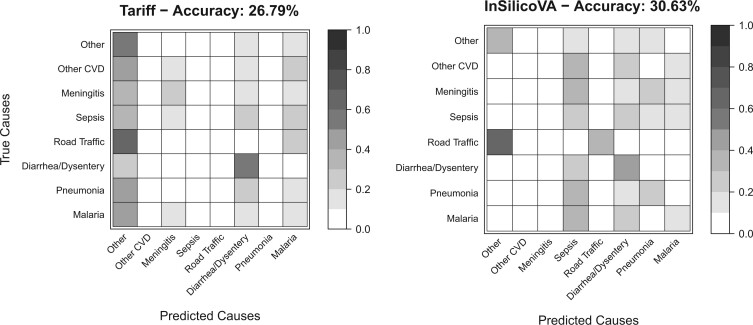
Confusion matrices for PHMRC child cases in Tanzania using Tariff and InSilicoVA trained on all cases outside of Tanzania. CVD, cardio-vascular diseases.

Lack of sufficient labeled target-domain data implies that CCVA classifiers need to be trained on non-local data like the publicly available Population Health Metrics Research Consortium (PHMRC) Gold Standard VA database (Murray *and others*, 2011a), that has more than $10\,000$ paired physician and VA assessments of cause of death across four countries. However, there exists considerable skepticism about the utility of CCVA trained on non-local data (McCormick *and others*, 2016; Flaxman *and others*, 2018). To illustrate the issue, in Figure 1, we plot the confusion matrices between the true COD of the PHMRC child cases in Tanzania against the predicted COD for these cases using two CCVA algorithms, Tariff and InSilicoVA, both trained on all PHMRC child data non-local to Tanzania. Both matrices reveal very large transfer errors, some as high as $60\%$ indicating that the naive estimates of population-level class probabilities from CCVA classifiers trained on non-local source data are likely to be inaccurate thereby highlighting the need for transfer learning in this application. Additionally, like for any other application area, there exists considerable disagreement about which CCVA algorithm is the most accurate (Leitao *and others*, 2014; McCormick *and others*, 2016; Flaxman *and others*, 2018). In our experience, no method is universally superior, and a robust ensemble transfer learning approach guarding against use of inaccurate classifiers is desirable.

## 2. Transfer learning for population-level class probabilities

### 2.1. Naive approach

Let ${\mathbf p}= (p_1,p_2,\ldots,p_C)'$ denote the true population-level class probabilities in a target domain where we have abundant unlabeled covariate data, which we denote by $\mathcal U$, and a very small labeled data $\mathcal L$ of paired labels $G$ and covariates ${\mathbf s}$. Our estimand is $p_i=P(G=i)$ where $G$ denote the true (gold standard) class-membership. For a case $r$ with covariate ${\mathbf s}_r$, let $A_r=\mathcal A({\mathbf s}_r \;|\; {\mathcal G})$ denote the predicted class membership, based on ${\mathbf s}_r$, from the baseline classification algorithm $\mathcal A$ trained on some large “gold-standard” information ${\mathcal G}$. Our methodology is agnostic to the type of ${\mathcal G}$, it can be either a labeled data set from the source-domain or set of context-specific information (e.g., medical guidelines for determining COD) used to construct the classifier. If we do not use any transfer learning, the naive estimate of ${\mathbf p}$ from $\mathcal A$ is given by

(2.1) \begin{equation*}\label{eq:base} \widehat {\mathbf q} = (\hat q_1,\ldots,\hat q_C)', \mbox{ where } \hat q_i = \sum_{r \in \mathcal U} I(A_r=i) / N = v_i / N, \end{equation*}

where $v_i$ is the number of observations in $\mathcal U$ classified by $\mathcal A$ to category $i$, and $N=\sum_i v_i$ is the sample size of $\mathcal U$. If $\mathcal U$ is large enough to be representative of the target population, it is clear that

$$\widehat {\mathbf q} \approx {\mathbf q}=(q_1,\ldots,q_C)' \mbox{ where } q_i = \int_{{\mathbf s} \in \mathcal U} \mathcal A({\mathbf s} \;|\; {\mathcal G}) {\rm d}P({\mathbf s}) = P(A = i), $$

i.e., $\widehat {\mathbf q}$ is the method-of-moments estimator of ${\mathbf q}$.

Unless the algorithm $\mathcal A$ trained on source-domain information perfectly agrees with the true membership assignment mechanism $G$ in the target domain, there is no reason to consider ${\mathbf q}$ or $\widehat {\mathbf q}$ to be a good estimate of ${\mathbf p}$. More realistically, accuracy depends on how similar the algorithm $\mathcal A$ is in the source- and target-domains. Hence, more generally we can think of ${\mathbf q}$ as the expected population class probabilities in the target domain that would be predicted by $\mathcal A(\cdot \;|\; {\mathcal G})$.

Marginally, both $G$ and $A$ are categorical variables. So, we can write

(2.2) \begin{equation*}\label{eq:pop_csmf} A \sim \mbox{Multinomial} (1,{\mathbf q}), \, G \sim \mbox{Multinomial} (1,{\mathbf p}). \end{equation*}

To infer about $G$ from $A$, we need to model their joint distributions. We express $q_j = \sum_{i=1}^C m_{ij} p_i,$ where $m_{ij} = p (A = j \;|\; G =i) $. In matrix notation, we have ${\mathbf q}={\mathbf M}'{\mathbf p}$ where ${\mathbf M}=(m_{ij})$ is a transition matrix (i.e., ${\mathbf M}\mathbf 1=\mathbf 1$) which we refer to as the *confusion matrix*. First note that, if ${\mathbf M}=\mathbf I$, then ${\mathbf p}={\mathbf q}$ and hence this subsumes the case where class probabilities from the baseline algorithm is trusted as reliable surrogates of the true class probabilities.

For transfer learning to improve estimation of ${\mathbf p}$, instead of assuming ${\mathbf M}=\mathbf I$, we can opt to use the more general relationship ${\mathbf q}={\mathbf M}'{\mathbf p}$ and estimate the transfer error rates $m_{ij}$’s from $\mathcal L$. Let $n = \sum_{i=1}^C n_i$ denote the sample size of $\mathcal L$ with $n_i$ denoting the number of objects belonging to class $i$. Also let

$$ \mathbf T=(t_{ij})=\sum_{r \in \mathcal L} I(A_r=j \;|\; G_r=i) $$

denote the *transfer error matrix* for algorithm $\mathcal A$. Like many transfer learning algorithms, exploiting the transfer errors is key to our strategy. It is clear that $t_{ij}/n_i$ is a method-of-moments estimator of $m_{ij}$.

We can use these estimates of $m_{ij}$, along with the earlier estimate of ${\mathbf q}$ to obtain a substantially improved estimate of ${\mathbf p}$. Formally we can specify this via a hierarchical model as:

(2.3) \begin{equation*}\label{eq:calib} \begin{array}{cc} A_r {\overset{iid}{\sim}}& \mbox{Multinomial} (1, {\mathbf M}'{\mathbf p}), r=1,2,\ldots,N \\ \mathbf T_{i*} \overset{ind}{\sim}& \mbox{Multinomial} (n_i, {\mathbf M}_{i*}), i=1,2,\ldots,C, \end{array} \end{equation*}

where for $r=1,2,\ldots,N$, and for any matrix ${\mathbf M}$, ${\mathbf M}_{i*}$ and ${\mathbf M}_{*j}$ denote its $i$th row and $j$th column respectively. The top-row of (2.3) represents the relationship ${\mathbf q} = {\mathbf M}'{\mathbf p}$ and yields the method-of-moments estimators $\widehat{\mathbf q} = (v_1,v_2,\ldots,v_C)'/N$. The bottom-row of (2.3) is consistent with the naive estimates $t_{ij}/n_i$ of $m_{ij}$.

To estimate ${\mathbf p}$, one can adopt a modular two-step approach where first $\widehat{\mathbf q}$ and $\widehat{\mathbf M}$ are calculated separately and then obtain

$$ \widehat {\mathbf p} = \underset{{\mathbf p}: \mathbf 1'{\mathbf p} =1,\; p_i \geq 0} {\arg \min} L(\widehat {\mathbf q}, \widehat {\mathbf M}'{\mathbf p}),$$

where $L$ is some loss function like the squared-error or, more appropriately, the Kullback–Liebler divergence between the probability vectors. This approach fails to propagate the uncertainty in the estimation of ${\mathbf M}$ in the final estimates of ${\mathbf p}$. Benefits of a one-stage approach over a two-stage one has been demonstrated in recent work in transfer learning (Long *and others*, 2014). Alternatively, one can estimate the joint MLE of ${\mathbf M}$ and ${\mathbf p}$ from (2.3).

The advantage of this simple transfer learning method is that it circumvents the need to improve the individual predictions of $\mathcal A$ and directly calibrates the population-level class probabilities ${\mathbf p}$, which are the quantities of interest here. We deliberately model the distribution of $(A,G)$ marginalized over the covariates ${\mathbf s}$, allowing us to use this in situations where the covariates are discarded after predictions or cannot be provided due to privacy concerns, and only the predictions $A_r$ are available. Even if the covariates were available, trying to use the extremely small $\mathcal L$ to estimate $P({\mathbf s} \;|\; G)$ relationship is not advisable. Instead, we efficiently exploit the small local training data $\mathcal L$ to reduce cross-domain bias learning about the ($P(A \;|\; G)$) confusion matrix. This involves estimation of only $C(C-1)$ parameters as opposed to the $\mathcal O(SC)$ parameters if trying to estimate $P({\mathbf s} \;|\; G)$. For verbal autopsy data, $S$ is typically around $250$ while we can choose $C$ to be small focusing on the top 3–5 causes. Hence, our approach achieves considerable dimension reduction by switching from the original covariate space to the predicted class space.

In (2.3) above, ${\mathbf q}={\mathbf M}'{\mathbf p}$ can be directly estimated precisely because $N$ is large. However, ${\mathbf M}$ has $C\times (C-1)$ parameters so that if there are many classes, the estimates of $m_{ij}$ will have large variances owing to the small size of $\mathcal L$. Furthermore, in epidemiological studies, data collection often spans a few years; in the early stages, $\mathcal L$ may only have a very small sample size resulting in an extremely imprecise estimate of ${\mathbf M}$, even if we group the classes to a handful of larger classes. This can lead to an imprecise estimate of ${\mathbf p}$ based on estimates of ${\mathbf q}$ and ${\mathbf M}$, as the latter will have high variance. Consequently, in the next section we propose a regularized approach that stabilizes the transfer learning.

### 2.2. Bayesian regularized approach

We champion the idea that in absence of enough target-domain labeled data, our method should shrink towards the default method familiar to practitioners. If $\mathcal L$ was not available, i.e., there is no labeled data in the target domain, we only have $\mathcal U$ and ${\mathcal G}$. Then in many applications (including verbal autopsies) the current practice is to train $\mathcal A$ once using ${\mathcal G}$ and then predict on $\mathcal U$ to obtain $\widehat {\mathbf q}$ as the estimate for ${\mathbf p}$. This is equivalent to setting ${\mathbf p} = {\mathbf q}$ and ${\mathbf M}=\mathbf I$, i.e., assuming that the algorithm $\mathcal A$ perfectly classifies in the target domain even when trained only using source-domain information ${\mathcal G}$. Extending this argument, when $\mathcal L$ is very small, direct estimates of ${\mathbf M}$ would be unstable and we can rely more on this default practice. Hence, for small $\mathcal L$, we will shrink ${\mathbf p}$ towards ${\mathbf q}$ i.e., we shrink towards the default assumption that the baseline learner is accurate. This is equivalent to shrinking the estimate of ${\mathbf M}$ towards $\mathbf I$. The simplest way to achieve this is by using the regularized estimate $\widetilde {\mathbf M} = (1- \lambda) \widehat {\mathbf M} + \lambda \mathbf I,$ where $\widehat {\mathbf M} = (\widehat m_{ij}) = t_{ij} / n_i$ is the unshrunk method-of-moments estimate of $m_{ij}$ as derived in the previous section. The regularized estimate $\widetilde {\mathbf M}$ (like $\widehat{\mathbf M}$ and ${\mathbf M}$) remains a transition matrix. The parameter $\lambda$ quantifies the degree of shrinkage with $\lambda=0$ yielding the unbiased method-of-moments estimate and $\lambda=1$ leading to $\widehat {\mathbf p} = \widehat {\mathbf q}$. Hence, $\lambda$ represents the bias variance trade-off for estimation of transition matrices and for small sample sizes some intermediate values of $\lambda$ may lead to better estimates of ${\mathbf M}$ and ${\mathbf p}$.

In epidemiological applications, as data will often come in batches over a period spanning few years, one needs to rerun the transfer learning procedure periodically to update the class probabilities. In the beginning, when $\mathcal L$ is extremely small, it is expected that more regularization is required. Eventually, when $\mathcal L$ becomes large, we could rely on the direct estimate $\widehat {\mathbf M}$. Hence, $\lambda$ should be a function of the size $n$ of $\mathcal L$, with $\lambda = 1$ for $n=0$ and $\lambda \approx 0$ for large $n$. Furthermore, at intermediate stages, since the distribution of true class memberships in $\mathcal L$ will be non-uniform across the classes, we will have a disparity in sample sizes $n_i$ for estimating the different rows of ${\mathbf M}$. Consequently, it makes more sense to regularize each row of ${\mathbf M}$ separately instead of using a single $\lambda$. A more flexible regularized estimate is given by $\widetilde {\mathbf M}_{i*} = (1 - \lambda_i) \widehat {\mathbf M}_{i*} + \lambda_i \mathbf I_{i*}$. The row specific weights $\lambda_i$ should be chosen such that $\lambda_i = 1$ when $n_i = \sum_{j=1}^C t_{ij} = 0$, and $\lambda_i \approx 0$ when $n_i$ is large. One choice is given by $\lambda_i = \gamma_i / (n_i + \gamma_i)$ for some fixed positive $\gamma_i$’s.

We now propose a hierarchical Bayesian formulation that accomplishes this regularized estimation of any transition matrix ${\mathbf M}$. We consider a Dirichlet prior ${\mathbf M}_{i*} \overset{ind}{\sim} \mbox{Dirichlet} (\gamma_i (\mathbf I_{i*} + \epsilon \mathbf 1))$ for the rows of ${\mathbf M}$. We first offer some heuristics expounding choice of this prior. We will have ${\mathbf M}_{i*} \;|\; \mathbf T_{i*}, \gamma_i \sim$ Dirichlet$(\mathbf T_{i*} + \gamma_i (\mathbf I_{i*} + \epsilon 1))$. Hence,

$$E({\mathbf M}_{i*} \;|\; \mathbf T_{i*}, \gamma_i) = \frac {\mathbf T_{i*} + \gamma_i (\mathbf I_{i*} + \epsilon \mathbf 1) }{n_i + \gamma_i (1 + C\epsilon)} \overset{\epsilon \rightarrow 0}{\rightarrow} (1 - \lambda_i) \frac {\mathbf T_{i*}}{n_i} + \lambda_i \mathbf I_{i*}, \mbox{ where } \lambda_i = \frac{\gamma_i}{n_i + \gamma_i} .$$

Hence, using a small enough $\epsilon$, the Bayes estimator (posterior mean) for ${\mathbf M}$ becomes equivalent with the desired shrinkage estimator $\widetilde {\mathbf M}_{i*}$ proposed above. When $n=0$, the Bayes estimate $E({\mathbf M} \;|\; \mathbf T, \boldsymbol \gamma=(\gamma_1,\gamma_2, \ldots, \gamma_C)') \approx \mathbf I$, and for large $n$, $E({\mathbf M} \;|\; \mathbf T, \boldsymbol \gamma)$ becomes the method-of-moments estimator $\widehat{\mathbf M}$. Hence, the Dirichlet prior ensures that in data-scarce setting, ${\mathbf M}$ is shrunk towards $\mathbf I$ and consequently ${\mathbf p}$ towards ${\mathbf q}$. In Theorem 2.1, we will present a more formal result that looks at the properties of the marginal posterior of ${\mathbf p}$.

To complete the hierarchical formulation, we augment (2.3) with the priors:

(2.4) \begin{equation*}\label{eq:hier} \begin{array}{rl} {\mathbf M}_{i*} \overset{ind}{\sim}& \mbox{Dirichlet} (\gamma_i (\mathbf I_{i*} + \epsilon \mathbf 1)), i=1,2,\ldots,C \\ {\mathbf p} \sim& \mbox{Dirichlet} (\delta \mathbf 1) \\ \gamma_i \overset{ind}{\sim}& Gamma(\alpha,\beta), i=1,2,\ldots,C \end{array} \end{equation*}

In practice, we need to use a small $\epsilon > 0$ to ensure a proper posterior for ${\mathbf M}$ when any off-diagonal entries of $\mathbf T$ are zero, which is very likely due to the limited size of $\mathcal L$. Note that our model only uses the data from $\mathcal L$ to estimate the conditional probabilities $P(A \;|\; G)$. We do not model the marginal distribution of $A$ or $G$ in $\mathcal L$ like we do for $\mathcal U$. This is because often data for the labeled set are collected under controlled settings, and marginal distribution of $G$ (and hence of $A$) for the samples in $\mathcal L$ is not representative of their true target-domain marginal distributions. Hence, we only use $\mathcal L$ to estimate the conditional probabilities ${\mathbf M}$.

Our previous heuristic arguments, illustrating the shrinkage estimation of ${\mathbf M}$ induced by the Dirichlet prior, are limited to the estimation of ${\mathbf M}$ from $\mathcal L$ as an independent piece and disregards the data and model for $\mathcal U$, i.e. the first row of (2.3). In a hierarchical setup, however, the models for $\mathcal U$ and $\mathcal L$ contribute jointly to the estimation of ${\mathbf M}$ and ${\mathbf p}$. We will now state a more general result that argues that for our full hierarchical model specified through (2.3) and (2.4), when there is no labeled target-domain data or if the algorithm $\mathcal A$ demonstrates perfect accuracy (zero transfer error) on $\mathcal L$, then the marginal posterior estimates of ${\mathbf p}$ from our model coincides with the baseline estimates $\widehat{\mathbf q}$. Before stating the result, first note that the likelihood for $\mathbf a=(A_1,A_2,\ldots,A_N)'$ can be represented using the sufficient statistics $\mathbf v=(v_1,v_2,\ldots,v_C)'$. We can write $p(\mathbf a) \propto \prod_{j=1}^C q_j^{v_j}$ and hence ${\mathbf p},{\mathbf M},\boldsymbol \gamma | \mbox{data} = {\mathbf p},{\mathbf M},\boldsymbol \gamma \;|\; \mathbf v, \mathbf T$.

THEOREM 2.1 If $\mathbf T$ is a diagonal matrix, i.e., either there is no $\mathcal L$, or $\mathcal A$ classifies perfectly on $\mathcal L$, then $\lim_{\epsilon \to 0} {\mathbf p} \;|\; \mathbf v, \mathbf T \sim {\rm Dirichlet}(\mathbf v+\delta \mathbf 1)$. For $\delta=0$, $\lim_{\epsilon \to 0} E({\mathbf p} \;|\; \mathbf v, \mathbf T) = \widehat {\mathbf q}$.

The proofs of the theorems are provided in Section S4 of the Supplementary Materials available at *Biostatistics* online. Note that Theorem 2.1 is a result about the posterior of our quantity of interest ${\mathbf p}$, marginalizing out the other parameters ${\mathbf M}$, and the $\gamma_i$’s from the hierarchical model specified through (2.3) and (2.4). We also highlight that this is not an asymptotic result and holds true for any sample size as long as we take the limit $\epsilon \rightarrow 0$. This is important as our manuscript pertains to epidemiological applications where the sample size of $\mathcal L$ will be extremely small.

Theorem 2.1 also does not require any assumption about the underlying data generation scheme and is simply a desirable property of our transfer learning model. If there is no labeled target-domain data, then we give the same estimate from the method currently used by practitioners, i.e., we trust $\mathcal A$ trained on a source-domain and only use the target-domain marginal distributions of ${\mathbf s}$ from $\mathcal U$ to arrive at the estimates $\widehat {\mathbf q}$ of ${\mathbf p}$. Similarly, in the best-case scenario, when $\mathcal A$ is absolutely accurate for the target domain, Theorem 2.1 guarantees that our model automatically recognizes this accuracy and does not modify the baseline estimates $\widehat {\mathbf q}$ from $\mathcal A$. This shrinkage towards the default method familiar to practitioners, in absence of enough evidence, is a desirable property. The result of Theorem 2.1 is confirmed in simulations in Section 5.

Although Theorem 2.1 is assumption-free, it only concerns with the performance of the model when there is no $\mathcal L$ or when $\mathcal A$ is perfect on $\mathcal L$. While this is a good sanity check for our model, realistically we will have a small $\mathcal L$ where $\mathcal A$ will be inaccurate. In such cases, the performance of our model will of course depend on the data generation process. Hence, we summarize the data generation assumption that drive the model formulation. Since, there is no labeled data in $\mathcal U$, we need to assume some commonality between $\mathcal L$ and $\mathcal U$ in order for the labeled data in $\mathcal L$ to be useful for estimating the CSMFs in $\mathcal U$. Hence, the model assumes that the conditional distribution of $A \;|\; G$ (i.e., the ${\mathbf M}$ matrix) is same in $\mathcal U$ and $\mathcal L$. This is a *transportability assumption* that the error rates on the validation set $\mathcal L$ reflects the true error rates in the population $\mathcal U$. These rates are then used to correct for bias in the estimates of ${\mathbf p}$. This process is thus analogous to applying measurement error correction to a study by assuming transportability of the measurement error distribution from some validation samples (Carroll *and others*, 2006). We would like to emphasize that we do not assume that the marginal distributions of the cause $G$ (i.e., the CSMFs) and hence also of the symptoms ${\mathbf s}$ are same in any of ${\mathcal G}$, $\mathcal U$ and $\mathcal L$. Of course, the assumption of same confusion matrix ${\mathbf M}$ for $\mathcal U$ and $\mathcal L$ can also be incorrect (all models are wrong). However, the class of models spanned by use of a general ${\mathbf M}$ is a superset of the default approach of using the baseline classifier (i.e., assuming ${\mathbf M}=\mathbf I$). Also, we can relax the assumption of constant ${\mathbf M}$ between $\mathcal U$ and $\mathcal L$ to make entries of ${\mathbf M}$ function of some covariates. This model and its implementation is discussed in Section 4. This would lead to substantial increase in parameter dimensionality and is only recommended when $\mathcal L$ is large.

### 2.3. Gibbs sampler using augmented data

We devise an efficient implementation of the hierarchical transfer learning model using a data augmented Gibbs sampler. The joint posterior density can be expressed as

$$
p({\mathbf p}, {\mathbf M}, \boldsymbol \gamma \;|\; \mathbf v, \mathbf T) \propto \; p(\mathbf v \;|\; {\mathbf M}, {\mathbf p})p(\mathbf T \;|\; {\mathbf M})p({\mathbf M} \;|\; \boldsymbol \gamma)p({\mathbf p})p(\boldsymbol \gamma).$$

Let ${\mathbf p} \;|\; \cdot$ denote the full conditional distribution of ${\mathbf p}$. We use similar notation for other full conditionals. First note that since $p(\mathbf v \;|\; {\mathbf M}, {\mathbf p}) \propto \prod_j (\sum_i m_{ij}p_i)^{v_j}$, the full conditional densities ${\mathbf p} \;|\; \cdot$ and ${\mathbf M} \;|\; \cdot$ do not belong to any standard family of distributions, thereby prohibiting a direct Gibbs sampler. We here use a data augmentation scheme enabling a Gibbs sampler using conjugate distributions.

The term $(\sum_{i} m_{ij} p_{i})^{v_j}$ can be expanded using the multinomial theorem, with each term corresponding to one of the partitions of $v_j$ into $C$ non-negative integers. Equivalently we can write

$$
\left(\sum_{i} m_{ij} p_{i}\right)^{v_j} \propto E \left(\prod_{i} (m_{ij}p_i)^{b_{ij}}\right) \mbox{ where } \mathbf b_j =(b_{1j},\ldots,b_{Cj})'\sim \mbox{ Multinomial}(v_j,\mathbf 1/C).$$

Choosing $\mathbf b_1, \mathbf b_2, \ldots, \mathbf b_C$ to be independent, we can express $\prod_{j} (\sum_{i} m_{ij} p_{i})^{v_j} \propto E(\prod_j \prod_i (m_{ij}p_i)^{b_{ij}} )$ where the proportionality constant only depends on the observed $v_j$’s. Using the augmented data matrix $\mathbf B= (\mathbf b_1, \mathbf b_2, \ldots, \mathbf b_C) = (b_{ij})$, we can write the complete posterior as

(2.5) \begin{equation*}\label{eq:postaug} \begin{array}{cc} p({\mathbf p}, {\mathbf M}, \boldsymbol \gamma, \mathbf B \;|\; \mathbf v, \mathbf T) \propto & \prod_j \prod_i (m_{ij}p_i)^{b_{ij}} \times \prod_{i} p_{i}^{\delta - 1} \times \prod_i \gamma_i ^{\alpha-1} \exp(-\beta \gamma_i) \times \\ & \prod_{i} \left( \frac{\Gamma(C\gamma_i\epsilon + \gamma_i)}{\Gamma(\gamma_i\epsilon)^{C - 1}\Gamma(\gamma_i\epsilon + \gamma_i)} \prod_{j}(m_{ij})^{t_{ij} + \gamma_i\epsilon + \gamma_i1(i = j) - 1} \right)\!. \end{array} \end{equation*}

The full conditional distributions can be updated as follows (derivations omitted):

$$\begin{align*}
\mathbf b_j \;|\; \cdot &\sim {\rm Multinomial}(v_j, \frac 1{\sum_i m_{ij}p_i}( m_{1j}p_{1}, m_{2j}p_{2}, \ldots, m_{Cj}p_{C})')\\
{\mathbf M}_{i*} \;|\; \cdot &\sim {\rm Dirichlet}(b_{i1} + \gamma_i\epsilon + t_{i1}, \ldots, b_{ii} + \gamma_i\epsilon + t_{ii} + \gamma_i, \ldots, b_{iC} + \gamma_i\epsilon + t_{iC}) \\
{\mathbf p} \;|\; \cdot &\sim {\rm Dirichlet} \left(\sum_{j} b_{1j} + \delta, \ldots, \sum_{j} b_{Cj} + \delta\right) \\
p(\gamma_i \;|\; \cdot) &\propto \frac{\Gamma(C\gamma_i\epsilon + \gamma_i)}{\Gamma(\gamma_i\epsilon)^{C - 1}\Gamma(\gamma_i\epsilon + \gamma_i)} \gamma_i ^{\alpha-1} \exp(-\beta \gamma_i) \prod_{j}m_{ij}^{\gamma_i\epsilon + \gamma_i1(i = j) }.
\end{align*}$$

The data augmentation ensures that, except the $C$$\gamma_i$’s, which are updated using a metropolis random walk with log-normal proposals, all the other $\mathcal O(C^2)$ parameters are update by sampling from standard distributions leading to an extremely fast and efficient Gibbs sampler.

## 3. Ensemble transfer learning

Let there be $K$ classifiers $\mathcal A^{(1)}, \ldots, \mathcal A^{(K)}$ and let $\mathbf a^{(k)}=(a_1^{(k)}, a_2^{(k)}, \ldots, a_N^{(k)})'$ be the predicted class memberships from the $k$th algorithm for all the $N$ observations in $\mathcal U$. Let $\mathbf v^{(k)}$ denote the vector of counts of predicted class memberships on $\mathcal U$ using $\mathcal A^{(k)}$. We expect variation among the predictions from the different classifiers and consequently among the baseline estimates of population-level class probabilities $\widehat {\mathbf q}^{(k)} = \mathbf v^{(k)}/N$ and their population equivalents ${\mathbf q}^{(k)}=P(A^{(k)})$. Since the true population class probability vector ${\mathbf p}$ is unique, following Section 2.1 we can write ${\mathbf q}^{(k)} = (q_1^{(k)},q_2^{(k)},\ldots,q_C^{(k)})' = {\mathbf M}^{(k)'} {\mathbf p}$, where ${\mathbf M}^{(k)} = (m_{ij}^{(k)})$ is now the classifier-specific confusion matrix. The predicted class membership for the $r$th observation in $\mathcal U$ by algorithm $A^{(k)}$, denoted by $a_r^{(k)}$, marginally follows a $\mbox{Multinomial} (1,{\mathbf q}^{(k)})$ distribution. We have $K$ such predictions for the same observation, one for each classifier, and these are expected to be correlated. So, we need to look at the joint distribution of the $K$$C$-dimensional multinomial random variables. Since, in its most general form this will involve $\mathcal O(C^K)$ parameters, we use a pragmatic simplifying assumption to derive the joint distribution. We assume that $a_r^{(1)}, a_r^{(2)}, \ldots, a_r^{(K)}$ are independent conditional on $G_r$, i.e.,

(3.6) \begin{equation*}\label{eq:condind} p(a_r^{(1)}=j_1,a_r^{(2)}=j_2,\ldots,a_r^{(K)}=j_K \;|\; G_r=i) = \prod_{k=1}^K m_{ij_k}^{(k)}. \end{equation*}

This assumption is unlikely to hold in reality but is a common dimension reducing assumption used in classification problems. For example, the naive Bayes classifier uses this assumption to jointly model the probability of covariates given the true class memberships. Similar assumptions are used by InSilicoVA and InterVA to derive the joint distribution of the vector of symptoms ${\mathbf s}_r$. Here, we are applying the same assumption but not on ${\mathbf s}_r$ but on the lower-dimensional prediction vector $(a_r^{(1)}, a_r^{(2)}, \ldots, a_r^{(K)})'$.

Under this assumption, the marginal independence of the $a_r^{(k)}$’s will not generally hold. Instead we will have

(3.7) \begin{equation*}\label{eq:q} p(\mathbf a_r=\mathbf j) = p(a_r^{(1)}=j_1,a_r^{(2)}=j_2,\ldots,a_r^{(K)}=j_K) = \sum_{i=1}^C \left(\prod_{k=1}^K m_{ij_k}^{(k)}\right) p_i = w_{\mathbf j}, \end{equation*}

where $\mathbf j=(j_1,j_2,\ldots,j_K)$ denotes a $C\times 1$ vector index.

From the limited labeled data set $\mathcal L$ in the target domain, the classifier-specific transfer error matrices $\mathbf T^{(k)} = (t^{(k)}_{ij})=(\sum_{r \in \mathcal L} I(A^{(k)}_r=j \;|\; G_r = i))$ are also known and can be used to estimate the respective confusion matrices ${\mathbf M}^{(k)}$ in the same way ${\mathbf M}$ was estimated from $\mathbf T$ in Section 2.1. To introduce shrinkage in the estimation of ${\mathbf M}^{(k)}$, like in Section 2.2, we assign Dirichlet priors for each ${\mathbf M}^{(k)}$.

Let $\mathbf w$ denote a $C^K \times 1$ vector formed by stacking up all the $w_{j_1,j_2,\ldots,j_K}$’s defined in (3.7). The full specifications for the ensemble model that incorporates the predictions from all the algorithms is given by:

(3.8) \begin{equation*}\label{eq:enshier} \begin{array}{rl} \mathbf a_r \overset{iid}{\sim}& \mbox{Multinomial} (1,\mathbf w), r=1,2,\ldots,N \\ \mathbf T_{i*}^{(k)} \overset{ind}{\sim}& \mbox{Multinomial} (n_i, {\mathbf M}^{(k)}_{i*}), i=1,2,\ldots,C;\; k=1,2,\ldots,K \\ {\mathbf M}^{(k)}_{i*} \overset{ind}{\sim}& \mbox{Dirichlet} (\gamma^{(k)}_i (\mathbf I_{i*} + \epsilon \mathbf 1)), i=1,2,\ldots,C;\; k=1,2,\ldots,K \\ {\mathbf p} \sim& \mbox{Dirichlet} (\delta \mathbf 1) \\ \gamma^{(k)}_i \overset{ind}{\sim}& Gamma(\alpha,\beta), i=1,2,\ldots,C;\; k=1,2,\ldots,K \end{array} \end{equation*}

Although $\mathbf w$ is a $C^K \times 1$ vector, courtesy of the conditional independence assumption (3.6), it is only parameterized using the matrices ${\mathbf M}^{(k)}$ and ${\mathbf p}$ as specified in (3.7), and hence involves $KC^2+C$ parameters. This ensures that there is adequate data to estimate the enhanced number of parameters for this ensemble method, as for each ${\mathbf M}^{(k)}$ we observe the corresponding transfer error matrix $\mathbf T^{(k)}$. The Gibbs sampler for (3.8) is provided in Section S3 of the supplementary material available at *Biostatistics* online (http://www.biostatistics.oxfordjournals.org). To understand how the different classifiers are given importance based on their transfer errors on $\mathcal L$, we present the following result:

THEOREM 3.1 If $\mathbf T^{(1)}$ is diagonal with positive diagonal entries, and all entries of $\mathbf T^{(k)}$ are $\geq 1$ for all $k>1$, then ${\mathbf p} \;|\; {\rm data} \sim \mbox{Dirichlet } (\mathbf v^{(1)}+\delta)$. For $\delta=0$, $E({\mathbf p} \;|\; {\rm data}) = {\mathbf q}^{(1)}$.

Theorem 3.1 reveals that if one of the $K$ algorithms (which we assume to be the first algorithm without loss of generality) produce perfect prediction on $\mathcal L$, then posterior mean estimate of ${\mathbf p}$ from the ensemble model coincides with that of the baseline estimate from that classifier. The perfect agreement assumed in Theorem 3.1 will not occur in practice. However, simulation and data analyses will confirm that the estimate of ${\mathbf p}$ from the ensemble model tend to agree with that from the single-classifier model in Section 2.2 with the more accurate algorithm. This offers a more efficient way to weight the multiple algorithms, yielding a unified estimate of class probabilities that is more robust to inclusion of an inaccurate algorithm in the decision making. In comparison, a simple average of estimated ${\mathbf p}$’s from single-classifier transfer learning models for each of the $K$ algorithms would be more adversely affected by inaccurate algorithms.

### 3.1. Independent ensemble model

The likelihood for the top-row of (3.8) is proportional to $\prod_\mathbf j w_\mathbf j^{y_\mathbf j}$ where $y_\mathbf j=\sum_{r \in \mathcal U} I(a^{(1)}=j_1,\ldots,a^{(K)}_r=j_K)$ denote the total number of observations in $\mathcal U$ where the predicted class-memberships from the $K$ algorithms corresponds to the combination $\mathbf j=(j_1,\ldots,j_K)'$. Even though $\mathcal U$ will be moderately large (few thousand observations in most epidemiological applications), unless both $C$ and $K$ are very small ($C \leq 5$ and $K \leq 3$), $y_\mathbf j$’s will be zero for most of the $C^K$ possible combinations $\mathbf j$. This will in-turn affect the estimates of $\mathbf w$. For applications to verbal autopsy-based estimation of population CSMFs, there are many CCVA algorithms (as introduced in Section 1), and researchers often want to use all of them in an analysis. We also may be interested in more than 3–5 top causes. In such cases, the extremely sparse $C^K$ vector formed by stacking up the $\mathbf y_\mathbf j$’s will destabilize the estimation of $\mathbf w$. Also, the Gibbs sampler (see Section S3 of the supplementary material available at *Biostatistics* online) of the joint-ensemble model introduces an additional $C^K$ independent multinomial variables of dimension $C$ thereby accruing substantial computational overhead and entailing long runs of the high-dimensional Markov chain to achieve convergence.

In this section, we offer a pragmatic alternative model for ensemble transfer learning that is computationally less demanding. From (3.7), we note that

(3.9) \begin{equation*} p(a_r^{(k)} = j_k) = \sum_{j_s : s \neq k} \sum_{i=1}^C \left(\prod_{k=1}^K m_{ij_k}^{(k)}\right) p_i = \sum_{i=1}^C m_{ij_k}^{(k)} p_i \end{equation*}

by exchanging the summations. Hence, the marginal distribution of $a_r^{(k)}$ is $\mbox{Multinomial} (1, {\mathbf q}^{(k)}),$ where ${\mathbf q}^{(k)} = ({\mathbf M}^{(k)}) ' {\mathbf p}$. We model the $a_r^{(k)}$’s independently for each $k$, ignoring the correlation among the predictions in $\mathcal U$ from the $K$ classifiers as follows:

(3.10) \begin{equation*}\label{eq:ensind} \mathbf a_r=(a^{(1)}_r,a^{(2)}_r,\ldots,a^{(K)}_r)' \overset{iid}{\sim} \prod_{k=1}^K \mbox{Multinomial} (1, {\mathbf q}^{(k)}), r=1,\ldots,N \\ \end{equation*}

We replace the top-row of (3.8) with (3.10), keeping the other specification same as in (3.8). We call this the independent ensemble model. Note that, while we only use the marginal distributions of the $a_r^{(k)}$’s ignoring their joint dependence, the joint distribution is preserved in the model for the transfer errors on $\mathcal L$ specified in the second-row of (3.8), as all the ${\mathbf M}^{(k)}$’s are tied to the common truth ${\mathbf p}$ through the equations ${\mathbf q}^{(k)}={\mathbf M}^{(k)'}{\mathbf p}$. While the total number of parameters for the joint and independent ensemble models remain the same, eliminating the joint model for each of the $C^K$ combination of predicted causes from the $K$ algorithms allows decomposing the likelihood for (3.10) as product of individual likelihoods on $\mathcal U$ for each of the $K$ classifiers. Additionally, the Gibbs sampler for the independent ensemble model is much simpler and closely resembles the sampler for the single-classifier model in Section 2.3. We only need to introduce $K$$C\times C$ matrices $\mathbf B^{(k)}=(\mathbf b_1^{(k)}, \mathbf b_2^{(k)}, \ldots, \mathbf b_C^{(k)})$, one corresponding to each CCVA algorithm, akin to the matrix $\mathbf B$ introduced in Section 2.3. The Gibbs sampler steps for the independent ensemble model are:

$$\begin{align*}
\mathbf b_j^{(k)} \;|\; \cdot &\sim {\rm Multinomial}(v^{(k)}_j, \frac 1{\sum_i m^{(k)}_{ij}p_i}( m^{(k)}_{1j}p_{1}, m^{(k)}_{2j}p_{2}, \ldots m^{(k)}_{Cj}p_{C})')\\
{\mathbf M}^{(k)}_{i*} \;|\; \cdot &\sim {\rm Dirichlet}(\mathbf B^{(k)}_{i*}+\mathbf T^{(k)}_{i*}+\gamma^{(k)}_i \mathbf I_{i*} + \epsilon\boldsymbol \gamma^{(k)}_i \mathbf 1) \\
{\mathbf p} \;|\; \cdot &\sim {\rm Dirichlet}(\sum_k \sum_{j} b^{(k)}_{1j} + \delta, \ldots, \sum_k \sum_{j} b^{(k)}_{Cj} + \delta)
\end{align*}$$

Observe that the sampler for the independent model uses $CK$ additional parameters as opposed to $C^K$ parameters introduced in the joint sampler. This ensures that the Markov Chain dimensionality does not exponentially increase if predictions from more algorithms are included in the ensemble model. The theoretical result in Theorem 3.1 no longer remains true for the independent model. However, our simulation results in Section S5.5 of the supplementary material available at *Biostatistics* online (http://www.biostatistics.oxfordjournals.org) show that in practice it continues to put higher weights on the more accurate algorithm and consistently performs similar to or better than the joint model. In Section S1, we present an EM algorithm approach to obtain maximum a posteriori (MAP) estimates for the model as a fast alternative to the fully Bayesian approach adopted here. In Section S2, an algorithm for generating posterior samples of individual-level class predictions is outlined.

## 4. Demographic covariates and spatial information

The transfer-learning model introduced up to this point is focused on generating population-level estimates of the CSMF ${\mathbf p}$. An important extension for epidemiological applications would be to model ${\mathbf p}$ as a function of covariates like geographic region, seasonality, social economic status (SES), sex and age groups. This will enable the estimation of regional and age-sex stratified estimates. In this section, we generalize the model to accommodate covariates. We illustrate for the single-classifier model in Section 2.2; a similar approach extends the ensemble model.

Let $\mathbf x_r$ denote a vector of covariates for the $r^{th}$ VA record in $\mathcal U$. We propose the following modifications to the model for allowing covariate-specific class distributions ${\mathbf p}_r = (p_{r1},p_{r2},\ldots,p_{rC})'$:

(4.11) \begin{equation*}\label{eq:hiercov} \begin{array}{rl} A_r \overset{ind}{\sim}& \mbox{Multinomial} ({\mathbf M}'{\mathbf p}_{r}), r=1,2,\ldots,N \\ p_{ri} =& \frac{exp(\mathbf x_{r}'\boldsymbol \beta_{i})}{\sum_{i=1}^{C}exp(\mathbf x_{r}'\boldsymbol \beta_{i})}, i=1, 2, \ldots, C, \boldsymbol \beta_{C} = 0 \\ \boldsymbol \beta_i \overset{ind}{\sim}& N(\mathbf m_{0i}, \mathbf W_{0i}) \end{array} \end{equation*}

All other components of the original model in (2.3) and (2.4) remain unchanged. The middle row of (4.11) specify a multi-logistic model for the class probabilities using the covariates. The top row uses the covariate specific ${\mathbf p}_r$ to model the analogous class probabilities ${\mathbf q}_r = {\mathbf M}'{\mathbf p}_r$ as would be predicted by $\mathcal A$. Finally, the bottom row specifies Normal priors for the regression coefficients. The switch from a Dirichlet prior for ${\mathbf p}$ to the multi-logistic model implies we can no longer directly leverage conjugacy in the Gibbs sampler. Polson *and others* (2013) proposed a Polya-Gamma data augmentation scheme to allow conjugate sampling for generalized linear models. We now show how our own data augmentation scheme introduced in Section 2.3 harmonizes with the Polya-Gamma sampler to create a streamlined Gibbs sampler.

### 4.1. Gibbs sampler using Polya-Gamma scheme

We will assume there are $H$ unique combinations of covariate values—for example, if there are four geographic regions and three age groups, then $H=12$. If we have a continuous covariate, then $H=N$, where $N$ is the number of subjects sampled in $\mathcal U$. Then letting $h$, $h=1,\ldots,H$, represent a specific covariate combination $\mathbf x_h$, we can again represent the likelihood for $\mathbf a=(A_1,A_2,\ldots,A_N)'$ using the $H \times C$ sufficient statistics $\mathbf V=(v_{hj}),$ where $v_{hj}$ is the total number of subjects with covariate values $h$ that were predicted to have died of cause $j$. Let $\boldsymbol \beta = (\boldsymbol \beta_{1}, \boldsymbol \beta_{2}, \ldots, \boldsymbol \beta_{C-1})$. We now have

$$
p(\mathbf V \;|\; {\mathbf M}, \boldsymbol \beta) \propto \prod_{h=1}^{H}\prod_{j=1}^{C}(\sum_{i=1}^{C}m_{ij}p_{hi})^{v_{hj}}
$$

and the joint posterior density can now be expressed as

$$
p(\boldsymbol{\beta}, {\mathbf M}, \boldsymbol \gamma \;|\; \mathbf V, \mathbf T) \propto \; p(\mathbf V \;|\; {\mathbf M}, \boldsymbol{\beta})p(\mathbf T \;|\; {\mathbf M})p({\mathbf M} \;|\; \boldsymbol \gamma)p(\boldsymbol{\beta})p(\boldsymbol \gamma).$$

The terms that are different from Section 2.3 are $p(\mathbf V \;|\; {\mathbf M}, \boldsymbol{\beta})$ and $p(\boldsymbol{\beta})$. The sampling step for $\boldsymbol \gamma$ remains exactly the same as previously discussed. We will use a similar data augmentation strategy as in Section 2.3 and combine with a Polya-Gamma data augmentation to sample from this posterior distribution. We expand the term $(\sum_{i}m_{ij}p_{hi})^{v_{hj}} \propto E(\prod_{i}(m_{ij}p_{hi})^{b_{hij}})$ where

$$
\mathbf b_{hj} =(b_{h1j},\ldots,b_{hCj})'\overset{ind}{\sim} \mbox{ Multinomial}(v_{hj},\mathbf 1/C).$$

Let $\mathbf B$ denote the $HC \times C$ matrix formed by stacking all the $\mathbf b_{hj}$’s row-wise. We can write

$$
p(\boldsymbol{\beta}, \mathbf B, {\mathbf M}, \boldsymbol \gamma \;|\; \mathbf V, \mathbf T) \propto \; \prod_{h}\prod_{i}\prod_{j}(m_{ij}p_{hi})^{b_{hij}}\times p(\mathbf T \;|\; {\mathbf M})p({\mathbf M} \;|\; \boldsymbol \gamma)p(\boldsymbol{\beta})p(\boldsymbol \gamma).
$$

The following updates ensue immediately:

$$\begin{align*}
\mathbf b_{hj} \;|\; \cdot &\sim {\rm Multinomial}(v_{hj}, \frac 1{\sum_i m_{ij}p_{hi}}( m_{1j}p_{h1}, m_{2j}p_{h2}, \ldots, m_{Cj}p_{hC})')\\
{\mathbf M}_{i*} \;|\; \cdot & \sim {\rm Dirichlet} \left(\mathbf T_{i*} + \boldsymbol \gamma_i \mathbf I_{i*} + \boldsymbol \gamma_i \mathbf 1 + \left(\sum_{h}b_{hi1}, \ldots, \sum_{h}b_{hiC}\right)' \right)\!.
\end{align*}$$

For $\boldsymbol \beta_i$’s, we introduce the Polya-Gamma variables $\omega_{hi}$’s and define $\boldsymbol \Omega_{i} = \text{diag}(\{\omega_{hi}\}_{h=1}^{H})$, $n_{h} = \sum_{j}v_{hj}$, and $\boldsymbol \kappa_i = (\kappa_{1i}, \ldots, \kappa_{Hi})'$, where $\kappa_{hi} = \sum_{j}b_{hij} - n_{h} / 2$. Defining $\mathbf W_{i}^{-1} = \mathbf X'\boldsymbol \Omega_{i}\mathbf X + \mathbf W_{0i}^{-1}$, we then have

$$\begin{align*}
\omega_{hi} \;|\; \cdot &\sim PG(n_{h}, \mathbf x_{h}^{T}\boldsymbol \beta_{i} - c_{hi}) \mbox{ where } c_{hi}=\log\left(\sum_{k \neq i} exp(x_{h}^{T}\beta_{k})\right) \\
\boldsymbol \beta_{i} \;|\; \cdot &\sim \mathcal{N}(\mathbf m_{i}, \mathbf W_{i}) \mbox{ where } \mathbf m_{i}= \mathbf W_{i}\left(\mathbf X'(\boldsymbol \kappa_{i} - \boldsymbol \Omega_{i}\mathbf c_{i}) + \mathbf W_{0i}^{-1}\mathbf m_{0i}\right)\!.
\end{align*}$$

Here $PG$ denotes the Polya-Gamma distribution and $\mathbf c_i=(c_{1i},c_{2i},\ldots,c_{Hi})'$. This completes the steps of a Gibbs sampler where all the parameters except $\boldsymbol \gamma$ are updated via sampling from conjugate distributions. We can transform the posterior samples of $\boldsymbol{\beta}$ to obtain posterior samples of $p_{hi}$. Estimates of the marginal class distribution for the whole country can also be obtained by using the relationship $ p_{i} = \int p_{hi} {\rm d} P(h),$ where an empirical estimate of the covariate distribution $P(h)$ can be obtained from $\mathcal U$.

### 4.2. Covariate-specific transfer error

Until now, we have assumed that the transition matrix ${\mathbf M}$ is independent of the covariates. We can also introduce covariates in modeling the conditional probabilities $m_{ij}$’s using a similar multi-logistic regression. This model will be particularly useful if there is prior knowledge about covariate-dependent biases in the predictions from a classifier. Letting $m_{rij}$ denoting the conditional probabilities $p(A = j \;|\; G = i, \mathbf x_r),$ we can model

(4.12) \begin{equation*}\label{eq:mijcov} \begin{array}{rl} m_{rij} =& \frac{exp(\mathbf x_{r}'\boldsymbol \zeta_{ij})}{\sum_{i=1}^{C}exp(\mathbf x_{r}'\boldsymbol \zeta_{ij})}, i, j \in \{1, 2, \ldots, C\}, \boldsymbol \zeta_{iC} = 0 \\ \boldsymbol \zeta_{ij} \overset{ind}{\sim}& N(\mathbf m_{0ij}, \mathbf W_{0ij}), \; j < C. \end{array} \end{equation*}

The implementation will involve Polya-Gamma samplers for each row of ${\mathbf M}$ in a manner exactly similar to the sampler outlined above (we omit the details). Since we can only estimate the parameters $\boldsymbol \zeta_{ij}$ from the limited local data, we can only adopt this approach with a very small set of covariates for modeling the transfer error rates.

## 5. Simulation studies

The PHMRC study, conducted in four countries across six sites, is a benchmark database of paired VA records and GS-COD of children, neonates and adults. PHMRC data are frequently used to assess performance of CCVA algorithms. We conduct a set of simulation studies using the PHMRC data (obtained through the openVA package, version 1.0.5) to generate a wide range of plausible scenarios where the performance of our transfer learning models needs to be assessed with respect to the popular CCVA algorithms. First, we randomly split the PHMRC child data ($2064$ samples) into three parts representing ${\mathcal G}$, and initial $\mathcal L$ and initial $\mathcal U$ respectively using a 2:1:2 ratio, containing roughly $800$, $400,$ and $800$ samples, respectively. As accurate estimation of mortality fractions from most prevalent causes are usually the priority, we restrict our attention to four causes: the top three most prevalent causes in the target-domain data ($\mathcal L \cup \mathcal U$)—Pneumonia, Diarrhea/Dysentery, Sepsis, and an *Other* cause grouping together all the remaining causes.

We wanted to simulate scenarios where both (i) the marginal distributions $P(G)$ of the classes and (ii) the conditional distributions $P(A \;|\; G)$ are different between the source- and target-domains. To ensure the latter, given a confusion matrix ${\mathbf M} = (m_{ij})$ we want $P(A=j \;|\; G=i) = m_{ij}$ on $\mathcal L \cup \mathcal U$. We will achieve this by discarding the actual labels in $\mathcal L \cup \mathcal U$ and generating new labels such that an algorithm $\mathcal A$ trained on ${\mathcal G}$ shows transfer error rates quantified by ${\mathbf M}$ on $\mathcal L \cup \mathcal U$. Additionally, the new labels need to be assigned in a way to ensure that the target-domain class probability vector is ${\mathbf p}_\mathcal U$, for any choice of ${\mathbf p}_\mathcal U$ different from the source-domain class probabilities in ${\mathbf p}_{\mathcal G}$.

Note that if the true population class probabilities in the target domain needs to be ${\mathbf p}_\mathcal U$, then ${\mathbf q}_\mathcal U$, the population class probabilities as predicted by $\mathcal A$ is given by ${\mathbf q}_\mathcal U = {\mathbf M}' {\mathbf p}_\mathcal U$. Hence, we first use $\mathcal A$ trained on ${\mathcal G}$ to predict the labels for each case in the initial $\mathcal U$. We then resample cases from the initial $\mathcal U$ to create a final $\mathcal U$ such that the predicted labels of $\mathcal A$ has the marginal distribution ${\mathbf q}_\mathcal U$. Next, from Bayes theorem,

$$ p(G = i \;|\; A = j) = \frac{m_{ij}p_{\mathcal U,i}}{\sum_i m_{ij}p_{\mathcal U,i}} = \alpha_{ij}.$$

For cases in $\mathcal U$ such that $A=j$, we generate the new “true” labels from $\mbox{Multinomial} (1,(\alpha_{1j}, \alpha_{2j},\ldots,\alpha_{Cj})')$. This data generation process ensures that for any case in $\mathcal U$ both $G \sim \mbox{Multinomial} (1,{\mathbf p}_\mathcal U)$ and $A \;|\; G=i \sim \mbox{Multinomial} (1,{\mathbf M}_{i*})$ are approximately true. We repeat the procedure for $\mathcal L$, using the same ${\mathbf M}$ but a different ${\mathbf p}_\mathcal L$. This reflects the reality for verbal autopsy data where the symptom-given-cause dynamics is same for all deaths $\mathcal L \cup \mathcal U$ in the new country, but the hospital distribution of causes ${\mathbf p}_\mathcal L$ is unlikely to match the population CSMF ${\mathbf p}_\mathcal U$. For resampling to create the final $\mathcal L$, we also vary $n$—the size of $\mathcal L$ as $50$, $100$, $200,$ and $400$, to represent varying amount of local labeled that will be available at different stages of a project.

We consider two choices of $\mathcal A$: Tariff (version 1.0.3) and InSilicoVA (version 1.2.2). For ${\mathbf M}$, we use three choices. We have ${\mathbf M}_1=\mathbf I$,

$$\begin{equation*}
{\mathbf M}_2 = \left( \begin{array}{cccc}
1.00 & 0 & 0 & 0\\
0.65 & 0.35 & 0 & 0\\
0 & 0 & 0.5 & 0.5\\
0 & 0 & 0 & 1
\end{array} \right)
\end{equation*}$$

and ${\mathbf M}_3 = 0.6 * \mathbf I+ 0.1* \mathbf 1 \mathbf 1'$. The first choice represents the case where the algorithm $\mathcal A$ is perfect for predicting in the target domain. ${\mathbf M}_2$ with two large off-diagonal entries and all other off-diagonal ones being zero represents the scenario where there are one or two systematic sources of bias in $\mathcal A$ when trained on a source-domain different from the target domain. The specific choice of ${\mathbf M}_2$ depicts the scenario that $65\%$ of Diarrhea/Dysentery cases are classified as pneumonia and $50\%$ of sepsis deaths are categorized as some other cause. Finally, ${\mathbf M}_3$ represents the scenario where there are many small misclassifications.

To ensure that ${\mathbf p}_\mathcal U$ and ${\mathbf p}_\mathcal L$ are different, we generate pairs of probability vectors $({\mathbf p}_\mathcal L, {\mathbf p}_\mathcal U)'$ from Dirichlet$(\mathbf 1)$ distribution and divide the cases into three scenarios: *low:* CSMFA$({\mathbf p}_\mathcal L,{\mathbf p}_\mathcal U) < 0.4$, *medium:* $0.4<$ CSMFA$({\mathbf p}_\mathcal L,{\mathbf p}_\mathcal U) < 0.6$, and *high:* CSMFA$({\mathbf p}_\mathcal L,{\mathbf p}_\mathcal U) > 0.6$. Here, CSMFA denoting the CSMF accuracy is a metric quantifying the distance of a probability vector (${\mathbf p}_\mathcal L$) from a reference probability vector (${\mathbf p}_\mathcal U$) and is given by (Murray *and others*, 2011b):

$${\rm {CSMFA}}({\mathbf p}_\mathcal L, {\mathbf p}_\mathcal U) = 1 - \frac{||{\mathbf p}_\mathcal L - {\mathbf p}_\mathcal U||_1 }{2(1 - \min {\mathbf p}_\mathcal U)} .$$

**Fig. 2:**
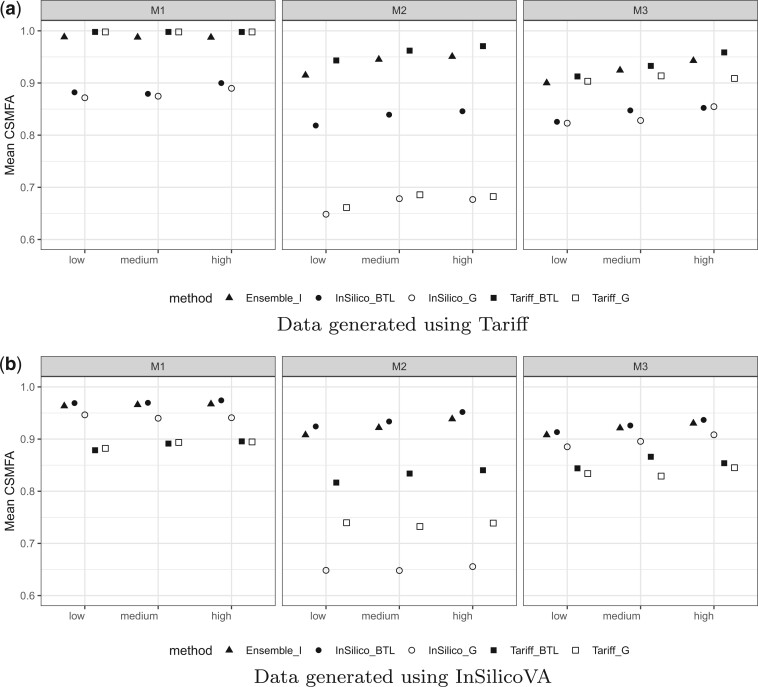
CSMF of ensemble and single-classifier transfer learners. (a) Data generated using Tariff. (b) Data generated using InSilicoVA.

For each scenario, we generated $100$ pairs of ${\mathbf p}_\mathcal L$ and ${\mathbf p}_\mathcal U$. For each generated dataset, we use all the algorithms listed in Table 2 for predicting ${\mathbf p}_\mathcal U$. For an estimate $\widehat {\mathbf p}_\mathcal U (x)$ generate by a model $x$, we assess the performance of $x$ using CSMFA$(x)$= CSMFA$(\widehat {\mathbf p}_\mathcal U (x), {\mathbf p}_\mathcal U)$. We present a brief summary of the results here. A much more detailed analysis is provided in Section S5 of the supplementary material (http://www.biostatistics.oxfordjournals.org). Figure 2 presents the CSMFA for all the five models for $n=400$.

**Table 2.** List of models used to estimate population CSMF

Model name	Description
Tariff$_{\mathcal G}$	Tariff trained on the source-domain gold standard data ${\mathcal G}$
$\mbox{Tariff}_{BTL}$	Bayesian transfer learner using the output from Tariff$_{\mathcal G}$
InSilico$_{\mathcal G}$	InSilicoVA trained on the source-domain gold standard data ${\mathcal G}$
$\mbox{InSilicoVA}_{BTL}$	Bayesian transfer learner using the output from InSilicoVA$_{\mathcal G}$
Ensemble$_{I}$	Ensemble Bayesian transfer learner (independent) using Tariff$_{\mathcal G}$ and Insilico$_{\mathcal G}$

The three columns are for the three choices of ${\mathbf M}$ described above, and in each figure the $x$-axis from left to right marks the *low, medium*, and *high* settings.

We observe that for almost all settings the Bayesian transfer learning approach was better than its corresponding baseline, i.e., $\mbox{Tariff}_{BTL}$ was better than $\mbox{Tariff}_{\mathcal G}$ and $\mbox{InSilicoVA}_{BTL}$ was better than $\mbox{InSilicoVA}_{\mathcal G}$. The improvement in CSMFA was most drastic for ${\mathbf M}_2$ (middle column) where it was as much as $0.3$ in some cases. Only for ${\mathbf M}_1$, i.e., when the classifier is assumed to be perfect for predicting in the target domain, we see $\mbox{Tariff}_{BTL}$ and $\mbox{Tariff}_{\mathcal G}$ produce similar CSMFA in the (top-left) and $\mbox{InSilicoVA}_{BTL}$ and $\mbox{InSilicoVA}_{\mathcal G}$ produce similar CSMFA (bottom-left). This just corroborates Theorem 2.1 that the transfer learning keeps things unchanged if the classifier has zero transfer error. We also observe that within each figure, CSMFA’s generally increase as we go from the low to the high setting, indicating that increased representativeness of the class distribution in the small labeled set $\mathcal L$ leads to improved performance. Also, across all settings we see that transfer learning based on algorithms used to simulate the data performs better, i.e., for the top-row $\mbox{Tariff}_{BTL}$ performs better than $\mbox{InSilicoVA}_{BTL}$ as in this case they respectively correspond to a true and a misspecified model. Similarly, for the bottom-row $\mbox{InSilicoVA}_{BTL}$ performs better than $\mbox{Tariff}_{BTL}$. However, even under model misspecification, the transfer learning models perform better than their baselines, i.e., even when data are generated using Tariff, $\mbox{InSilicoVA}_{BTL}$ performs better than $\mbox{InSilicoVA}_{\mathcal G}$. Finally, across all scenarios, the ensemble learner performs close to the better performing individual learner, highlighting its utility and robustness.

In Section S5 of the supplementary material available at *Biostatistics* online (http://www.biostatistics.oxfordjournals.org), we present more insights into the simulation study. Section S5.1 assesses the impact of the disparity in the class distributions between the source- and target-domains. In Section S5.2, we compare the biases in the estimates of individual class probabilities. Section S5.3 delves into the role of the sample size and quality of the limited labeled set $\mathcal L$. Section S5.4 demonstrates the value of the Bayesian shrinkage by comparing with the frequentist transfer learning outlined in Section 2.1. In Section S5.5, we compare the joint and independent ensemble models and demonstrate how they favorably weight the more accurate algorithm. Section S5.6 shows how one can use informed shrinkage, if a practitioner has *a priori* knowledge of which causes are likely to be misclassified by an algorithm. Finally, in Section S5.7, we compare the individual-level prediction performance of the models using the algorithm outlined in Section S5.2.

## 6. Predicting CSMF in India and Tanzania

We evaluate the performance of baseline CCVA algorithms and our transfer learning approach when predicting the CSMF for under 5 children in India and Tanzania using the PHMRC data with actual COD labels. We used both India and Tanzania, as they were the only countries with substantial enough sample sizes ($N_{\rm India} = 948$, $N_{\rm Tanzania} = 728$). For a given country (either India or Tanzania), we first split the PHMRC child data into subjects from within the country ($\mathcal L$ and $\mathcal U)$ and from outside of the country (${\mathcal G})$. We then used weighted sampling to select $n ( \in \{50, 100, 200\}$) subjects from within the country of interest to be in $\mathcal L$, using weights such that CSMFA$({\mathbf p}_{\mathcal L}, {\mathbf p}_\mathcal U)$ was low. Figure S8 in Section S6 of the supplementary material available at *Biostatistics* online (http://www.biostatistics.oxfordjournals.org) shows the difference in the marginal symptom distribution between $\mathcal U$ and $\mathcal L$. All the subjects from the country were put in $\mathcal U$. We trained models ${ Insilico_{G}}$ and ${Tariff_{G}}$ using the non-local data ${\mathcal G}$, which were then used to predict the top COD for all subjects in $\mathcal L$ and $\mathcal U$. We classified all causes of death into “External,” “Pneumonia,” “Diarrhea/Dysentery,” “Other Infectious,” and “Other.” These predictions were then used to estimate the baseline CSMFs and as an input to our transfer learning models $\mbox{Tariff}_{BTL}$, $\mbox{InSilicoVA}_{BTL}$, and ${\rm Ensemble_{I}}$. Since the true labels (GS-COD) are available in PHMRC, we calculated the true ${\mathbf p}_\mathcal U$ for a country as the empirical proportions of deaths from each cause, based on all the records within the country. This ${\mathbf p}_\mathcal U$ was used to calculate the CSMF accuracy of each model. This whole process was repeated $500$ times for each combination of country and value of $n$. This made sure that the results presented are average over $500$ different random samples of $\mathcal L$ for each country, and are not for an arbitrary sample.

**Fig. 3:**
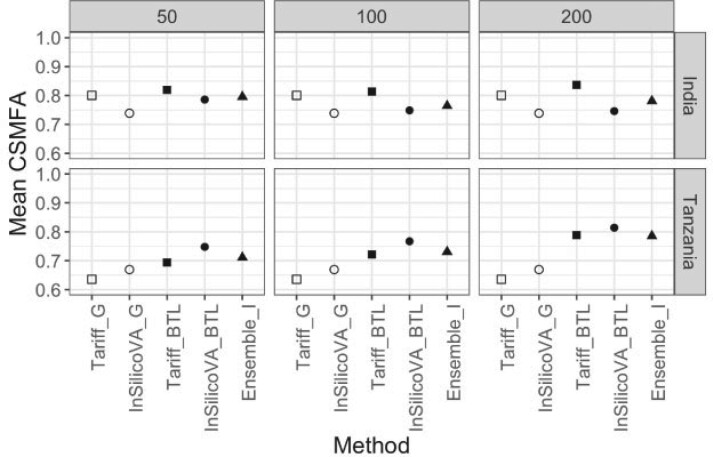
PHMRC analysis: average CSMFA using true GS-COD labels

Figure 3 presents the results of this analysis. The top and bottom rows represent the results for India and Tanzania, respectively. The four columns correspond to four different choices of $n$.

There are several notable observations. First, regardless of $n$, choice of algorithm $\mathcal A$, and country, the calibrated estimates of prevalence from our transfer learning model performed better than or similar to the analogous baseline CSMFs, i.e., $\mbox{Tariff}_{BTL}$ performed better than $\mbox{Tariff}_{\mathcal G}$, and $\mbox{InSilicoVA}_{BTL}$ performed better than $\mbox{InSilicoVA}_{\mathcal G}$. Second, the magnitude of improvement for the our approach depends on the country and the size of $\mathcal L$. Within India, the CSMFA of $\mbox{Tariff}_{BTL}$ and $\mbox{InSilicoVA}_{BTL}$ is similar to respectively those from $\mbox{Tariff}_{\mathcal G}$ and $\mbox{InSilicoVA}_{\mathcal G}$. Tariff does better than InsilicoVA for India with $\mbox{Tariff}_{BTL}$ being the best performer. In Tanzania, the baseline InsilicoVA model $\mbox{InSilicoVA}_{\mathcal G}$ does better than $\mbox{Tariff}_{\mathcal G}$, and similarly $\mbox{InSilicoVA}_{BTL}$ does better than $\mbox{InSilicoVA}_{BTL}$. The improvement of $\mbox{Tariff}_{BTL}$ and $\mbox{InSilicoVA}_{BTL}$, respectively over $\mbox{Tariff}_{\mathcal G}$ and $\mbox{InSilicoVA}_{\mathcal G}$ is more prominent than in India, with $\mbox{InSilicoVA}_{BTL}$ being the most accurate. The magnitude of improvement in the three TL approaches also increased with increase in $n$ for Tanzania. In Section S7 of the Supplementary material available at *Biostatistics* online, we investigate the effect of adding more causes of death on the transfer learning CSMFA.

## 7. Discussion

Epidemiological studies pose unique challenges to transfer learning, stemming from its focus on estimating population-level quantities as opposed to individual predictions, small sample sizes coupled with high-dimensional covariate spaces (survey records), and lack of large training databases available for many other machine learning tasks. Motivated by these settings, we have presented a parsimonious hierarchical model-based approach to transfer learning of population-level class probabilities, using a pre-trained classifier, limited labeled target-domain data, and abundant unlabeled target-domain data.

In order for the transfer learning approach to work, the labeled data $\mathcal L$ has to be useful for improving CSMF estimation in $\mathcal U$, i.e., there has to be some commonality between the distributions in $\mathcal L$ and $\mathcal U$. Usually $\mathcal L$ is not going to be representative of the marginal cause distribution of $\mathcal U$. If additionally, it is also not representative of the conditional distributions of ${\mathbf s} \;|\; G$ (or, equivalently by marginalizing out ${\mathbf s}$, of $A \;|\; G$), then $\mathcal L$ is of no use to improve CSMF estimation in $\mathcal U$. Hence, our transfer learning is useful when the conditional distributions are same (constant ${\mathbf M}$) between $\mathcal L$ and $\mathcal U$, or has the same functional form (regression approach of Section 4.2) between $\mathcal L$ and $\mathcal U$.

Shrinkage or regularization is at the core of our approach. In data sets with large numbers of variables (dimensions), regularized methods have become ubiquitous. A vast majority of the literature focuses on shrinking estimates (mostly regression coefficients and covariance or precision matrices) towards some known sub-model. We apply the same principle of regularization in a unique way for estimating the population class probabilities. Instead of shrinking towards any underlying assumptions about the true population distribution, we shrink towards the baseline estimate. In absence of sufficient target-domain data, this is the estimate that is commonly used, and this shrinkage principle is desirable for practitioners familiar with this default method. We show how this shrinkage is achieved by shrinking the confusion matrix towards the identity matrix using appropriate Dirichlet priors. This regularized estimation of a confusion matrix (or any transition matrix) can also be applied in other contexts.

The fully Bayesian implementation is fast, owing to a novel data-augmented Gibbs sampler. The ensemble model ensures robust estimates via data-driven averaging over many classifiers and reduces the risk of selecting a poor one for a particular application. Our simulations demonstrate the value of transfer learning, offering substantially improved accuracy. The PHMRC data analysis makes evident the value of collecting a limited number of labeled data GS-COD in the local population using full or minimally invasive autopsy, alongside the nationwide VA survey. Subsequently using transfer learning improves the CSMF estimates. The results also show how our approach benefits from larger sample sizes of the local labeled set $\mathcal L$, and from closer alignment between the marginal class probabilities in $\mathcal L$ and the true target-domain class probabilities.

For VA data analyses, we note that while we used a labeled data set in the source-domain as ${\mathcal G}$, in practice it can be any other form of gold standard information sufficient to train a VA classifier. CCVA methods like Tariff and the approach in King *and others* (2008) represent a traditional supervised learning approach and needs a substantial labeled training dataset ${\mathcal G}$. InterVA is a semi-supervised learning approach where ${\mathcal G}$ is a standard matrix of letter grades representing the propensity of each symptom given each cause. InSilicoVA generalizes InterVA and endows the problem with a proper probabilistic framework allowing coherent statistical inference. It adapts to the type of ${\mathcal G}$ and can work with either the default symptom-cause matrix used in InterVA or estimate this matrix based on some labeled training data of paired VA and GS-COD records. Our transfer learning is completely agnostic to the choice of this baseline CCVA algorithm and the form of ${\mathcal G}$ they require. We only need the predictions from a pre-trained algorithm for all observations in $\mathcal L \cup \mathcal U$.

One important direction forward would be to generalize this approach for more complex COD outcomes. Currently COD outcome is viewed as a discrete variable taking values on a set of causes like pneumonia or sepsis. In practice, death is a complex chronological series of several events starting from some root causes and ending at the immediate or proximal cause. In addition to understanding prevalence of causes in the population, another goal for many of the aforementioned programs is to identify medical events that occurred before death for which an intervention could prevent or delay mortality. Extending the current setup for hierarchical or tree-structured COD outcome would be a useful tool to address this aim. Many CCVA algorithms, in addition to predicting the most likely COD, also predict the (posterior) distribution of likely causes. Our current implementation only uses the most likely COD as an input. An extension enabling the use of the full predictive distribution as an input can improve the method. Finally, the VA records, containing about $250$ questions for thousands of individuals, naturally has several erroneous entries. Preprocessing VA records to eliminate absurd entries and records entails onerous manual efforts. It is challenging to develop quality control models for VA data due to the high dimensionality of the symptoms. Akin to what we did here, one can consider dimension reduction via the predictions of CCVA algorithms for automated statistical quality control.

## 8. Software

R-package “calibratedVA” containing code to obtain estimates of population CSMFs from our transfer learning approach using baseline predictions from any verbal autopsy algorithm is available at https://github.com/jfiksel/CalibratedVA/. The package also contains the code for the ensemble model for using outputs from several VA algorithms. A vignette describing how to navigate the package and demonstrating the use of the methodology is provided in https://github.com/jfiksel/CalibratedVA/blob/master/vignettes/CalibratedVA.Rmd. All results in this article can be recreated using the scripts contained in https://github.com/jfiksel/BayesTLScripts.

## Supplementary Material

kxaa001_Supplementary_Data
