# Supplementary material for: Regularized Bayesian transfer learning for population-level etiological distributions
Source: Biostatistics. 2020 Feb 10;22(4):836–57. doi: 10.1093/biostatistics/kxaa001 (PMC8511959; doi:10.1093/biostatistics/kxaa001)
Supplement: kxaa001_Supplementary_Data [file kxaa001_supplementary_data.pdf]

Biostatistics (2020), **0**, 0, *pp.* S1–S24
doi:10.1093/biostatistics/supplement

Supplementary material to Regularized Bayesian transfer learning for population-level etiological distributions

ABHIRUP DATTA*

Department of Biostatistics, Johns Hopkins University, 615 North Wolfe Street, Baltimore, MD 21205, USA

abhidatta@jhu.edu

JACOB FIKSEL

Department of Biostatistics, Johns Hopkins University, 615 North Wolfe Street, Baltimore, MD 21205, USA

AGBESSI AMOUZOU

Department of International Health, Johns Hopkins University, 615 North Wolfe Street, Baltimore, MD 21205, USA

SCOTT L. ZEGER

Department of Biostatistics, Johns Hopkins University, 615 North Wolfe Street, Baltimore, MD 21205, USA

S1. MAP estimation

In the main manuscript, we have only discussed fully Bayesian implementations of the model in (2.4). If full inferential output is superfluous and only posterior point-estimates of the parameters are desired, we outline a MAP (Maximum a posteriori) estimation for obtaining posterior modes

© The Author 2020. Published by Oxford University Press. All rights reserved. For permissions, please e-mail: journals.permissions@oup.com

of the parameters using an EM-algorithm. The data augmentation scheme introduced for the Gibbs sampler in 2.3 is also seamlessly congruous with the EM algorithm.

In particular, we consider the vector $\mathbf{v}$ and $\mathbf{T}$ as the observed data and augment $\mathbf{B}$ introduced in Section 2.3 as the missing data to form the complete data likelihood $l(\mathbf{B}, \mathbf{v}, \mathbf{T} \mid \mathbf{M}, \mathbf{p}, \boldsymbol{\gamma})$ which is proportional to (2.5). At the sth iteration, let $\mathbf{M}^{[s]} = (m_{ij}^{[s]})$, $\mathbf{p}^{[s]} = (p_i^{[s]})$ denote the current values of the parameters. Then

$$E^{[s]}(b_{ij} \mid \mathbf{v}, \mathbf{T}) = \frac{v_j m_{ij}^{[s]} p_i^{[s]}}{\sum_i m_{ij}^{[s]} p_i^{[s]}} = \widehat{b}_{ij}^{[s]},$$

where $E^{[s]}$ denotes the expectation taken using the parameter values from the sth iteration. The EM algorithm then proceeds as follows:

$$\begin{array}{c}\text{E-step: } E^{[s]}(\log l(\mathbf{B}, \mathbf{v}, \mathbf{T} \mid \mathbf{M}, \mathbf{p}, \boldsymbol{\gamma}) \mid \mathbf{v}, \mathbf{T}) = \sum_i \Bigg(\sum_j \Big(\widehat{b}_{ij}^{[s]} \log(m_{ij} p_i) + \\ (t_{ij} + \gamma_i \epsilon + \gamma_i I(i=j) - 1) \log(m_{ij}) \Big) + (\delta - 1) \log p_i + h(\gamma_i) \Bigg)\end{array} \tag{S1}$$

where $h(\gamma) = \log \left(\frac{\Gamma(C\gamma + \epsilon)}{\Gamma(\gamma\epsilon)^{C-1} \Gamma(\gamma\epsilon + \gamma)} \right) + (\alpha - 1) \log \gamma - \beta\gamma$. Subsequently, the maximization step can be formulated as:

$$\text{M-step:} \quad \begin{array}{rcl} m_{ij}^{[s+1]} & = & \frac{\widehat{b}_{ij}^{[s]} + t_{ij} + \gamma_i \epsilon + \gamma_i I(i=j) - 1}{\sum_j (\widehat{b}_{ij}^{[s]} + t_{ij}) + \gamma_i C\epsilon + \gamma_i - C} \\ p_i^{[s+1]} & = & \frac{\sum_j \widehat{b}_{ij}^{[s]} + \delta - 1}{\sum_i \sum_j \widehat{b}_{ij}^{[s]} + C\delta - C} \\ \gamma_i^{[s+1]} & = & \arg\max_\gamma \sum_j (\gamma\epsilon + \gamma I(i=j) - 1) \log(m_{ij}) + h(\gamma) \end{array} \tag{S2}$$

The closed form expression of $\mathbf{M}$ and $\mathbf{p}$ in the M-step is a consequence of the data augmentation. This drastically accelerates the MAP estimation as we only need to conduct C univariate optimizations, one corresponding to each γ_i. If instead the data augmentation was not exploited and only the observed likelihood was used, we would need to search an $O(C^2)$ dimensional space to obtain the MAP estimates. We can implement similar MAP estimation algorithms for the joint and independent ensemble models detailed in Section 3. We omit the steps here.

S2. Individual-level transfer learning

While our Bayesian transfer learning is primarily targeted to estimate population-level class probabilities, it can also be used to predict individual-level class probabilities in the target-domain. The posterior distribution of the true class membership G_r of the rth individual is given by

$$\begin{aligned}
p(G_r = i \mid A_r = j, \mathbf{T}) &= \int p(G_r = i \mid \mathbf{p}, \mathbf{M}, \boldsymbol{\gamma}, A_r = j, \mathbf{T}) \times \\
&\quad p(\mathbf{p}, \mathbf{M}, \boldsymbol{\gamma} \mid \mathbf{v}, \mathbf{T}) \; dP(\mathbf{M}) \; dP(\mathbf{p}) \; dP(\boldsymbol{\gamma}) \\
&= \int p(G_r = i \mid \mathbf{p}, \mathbf{M}, A_r = j) p(\mathbf{p}, \mathbf{M} \mid \mathbf{v}, \mathbf{T}) \; dP(\mathbf{M}) \; dP(\mathbf{p}) \\
&= \int \frac{m_{ij} p_i}{\sum_{i=1}^{C} m_{ij} p_i} p(\mathbf{p}, \mathbf{M} \mid \mathbf{v}, \mathbf{T}) \; dP(\mathbf{M}) \; dP(\mathbf{p})
\end{aligned}$$

We can now easily conduct composition sampling using posterior samples of $\mathbf{M}$ and $\mathbf{p}$ to generate a posterior distribution for G_r. This simple application of the Bayes theorem, can recover the individual class memberships. However, it is a crude approach because the posterior distribution of the G_r are identical for all instances with the same predicted class A_r from A. If A is a probabilistic classifier like InSilicoVA (McCormick *and others*, 2016), then in addition to providing a predicted class membership A_r, A also provides the predicted distribution for each individual's class. Utilizing the entire predicted distribution from A should lead to improved individual level transfer learning. Since the focus of this manuscript is population level transfer learning, we do not further explore this avenue here.

S3. Gibbs sampler for the joint ensemble model

Let $y_{\mathbf{j}}$ be the number of instances in $\mathcal{U}$ for which algorithm $A^{(1)}$ predicts cause j_1, $A^{(2)}$ predicts cause j_2, and so on. Let $\mathbf{y}^*$ be the $C^K \times 1$ vector formed by stacking the $y_{\mathbf{j}}$'s. Also, let $u_{i\mathbf{j}} = \prod_{k=1}^{K} m_{ij_k}^{(k)}$ and $\mathbf{u_j} = (u_{1\mathbf{j}}, u_{2\mathbf{j}}, \ldots, u_{C\mathbf{j}})'$.

The posterior $\mathbf{p}, \{\mathbf{M}^{(k)}, \boldsymbol{\gamma}_k\}_{k=1,\ldots,K} \mid \mathbf{T}^{(1)}, \mathbf{T}^{(2)}, \ldots, \mathbf{T}^{(k)}, \mathbf{w}^*$ is proportional to

$$\prod_{\mathbf{j}}(\sum_i u_{i\mathbf{j}}p_i)^{y_{\mathbf{j}}} \times \prod_i p_i^{\delta-1} \times$$
$$\prod_{k=1}^{K}\left(\prod_{i=1}^{C}\frac{\Gamma(\gamma_i^{(k)}(C\epsilon+1))}{(\Gamma(\gamma_i^{(k)}\epsilon))^{C-1}\Gamma(\gamma_i^{(k)}(\epsilon+1))}\times\prod_j (m_{ij}^{(k)})^{t_{ij}^{(k)}+\gamma_i^{(k)}(\epsilon+1(i=j))-1}\right).$$

We will once again use data augmentation to implement the Gibbs sampler. Let $\mathbf{b_j} = (b_{1\mathbf{j}}, b_{2\mathbf{j}}, \ldots, b_{C\mathbf{j}})')$ denote the $C \times 1$ dimensional realization of a Multinomial $(y_{\mathbf{j}}, \mathbf{1}/C)$ distribution, and let $\mathbf{B}$ denote the $C^K \times C$ matrix formed by stacking the independent $\mathbf{b_j}$'s row-wise for all combinations of $\mathbf{j}$. Then we have the following full conditionals for the Gibbs sampler:

$$\mathbf{b_j} \mid \cdot \sim Multinomial(y_{\mathbf{j}}, \frac{1}{\mathbf{1}'(\mathbf{u_j}\odot\mathbf{p})}\mathbf{u_j}\odot\mathbf{p})$$
$$\mathbf{M}_{i*}^{(k)} \mid \cdot \sim Dirichlet\left(\mathbf{T}_{i*} + \boldsymbol{\gamma}_i^{(k)}\mathbf{I}_{i*} + \boldsymbol{\gamma}_i^{(k)}\mathbf{1} + (\sum_{\mathbf{j}:j_k=1} b_{i\mathbf{j}}, \ldots, \sum_{\mathbf{j}:j_k=C} b_{i\mathbf{j}})'\right)$$
$$\mathbf{p} \mid \cdot \sim Dirichlet(\sum_{\mathbf{j}} b_{1\mathbf{j}} + \delta, \ldots, \sum_{\mathbf{j}} b_{C\mathbf{j}} + \delta)$$

Here $\odot$ denotes the Hadamard (elementwise) product.

Finally, as in Section 2.2, we update $\gamma_i^{(k)}$'s using a metropolis random walk with log-normal proposal to sample from the full conditionals

$$p(\gamma_i^{(k)} \mid \cdot) \propto \frac{\Gamma(C\gamma_i^{(k)}\epsilon+\gamma_i)}{\Gamma(\gamma_i^{(k)}\epsilon)^{C-1}\Gamma(\gamma_i^{(k)}\epsilon+\gamma_i^{(k)})}\times$$
$$(\gamma_i^{(k)})^{\alpha-1}\exp(-\beta\gamma_i^{(k)})\prod_j (m_{ij}^{(k)})^{\gamma_i^{(k)}\epsilon+\gamma_i^{(k)}1(i=j)}.$$

S3.1 *Individual level classifications*

As illustrated in Section S2, the ensemble transfer learner can also predict the individual-level class memberships. Using Bayes theorem we have

$$p(G_r = i \mid a_r^{(1)} = j_1, \ldots, a_r^{(K)} = j_k) = \frac{1}{\sum_{\mathbf{j}} u_{i\mathbf{j}}p_i}u_{i\mathbf{j}}p_i.$$

Since posterior distributions of $u_{i\mathbf{j}}$'s and $\mathbf{p}$ have already been sampled, we can generate posterior samples of G_r post-hoc using the composition sampling approach demonstrated in Section S2.

For the independent ensemble model, one can recover the posterior distribution of the individual class memberships in the exact same way. Only additional step would be to first calculate the $\mathbf{u_j}$'s as they are no longer part of the Gibbs sampler.

S4. Proofs

Theorem 2.1. The marginal posterior $\mathbf{p} \mid \mathbf{v}, \mathbf{T}$ is given by $\int p(\mathbf{p}, \mathbf{M}, \boldsymbol{\gamma} \mid \mathbf{v}, \mathbf{T}) dP(\mathbf{M}) dP(\boldsymbol{\gamma})$. Conditional on $\boldsymbol{\gamma}$, looking only at terms that involve $\mathbf{p}$, $\mathbf{M}$, and $\boldsymbol{\gamma}$, we have

$$p(\mathbf{p}, \mathbf{M}, \mathbf{v}, \mathbf{T} \mid \boldsymbol{\gamma}) \propto \prod_j (\sum_i m_{ij} p_i)^{v_j} \times \prod_i p_i^{\delta-1} \times \\ \prod_i \frac{\Gamma(\gamma_i(C\epsilon+1))}{(\Gamma(\gamma_i\epsilon))^{C-1}\Gamma(\gamma_i(\epsilon+1))} \prod_j (m_{ij})^{t_{ij}+\gamma_i(\epsilon+1(i=j))-1}$$

We will now use the multinomial theorem to expand the first product $\prod_j (\sum_i m_{ij} p_i)^{v_j}$. Note that the jth term expands into $\kappa_j = \binom{v_j+C-1}{C-1}$ terms, one corresponding to each partition of v_j. Let $\mathbf{B}^{(j)} = (b_{ki}^{(j)})$ denote the $\kappa_j \times C$ partition matrix formed by stacking up all $1 \times C$ rows that represent a non-negative integer partition of v_j. The kth row of $\mathbf{B}^{(j)}$ gives the kth partition and i^{th} element of that row corresponds to power index for the ith term $(m_{ij} p_i)$. We now have,

$$p(\mathbf{p}, \mathbf{M} \mid \mathbf{v}, \mathbf{T}, \boldsymbol{\gamma}) \propto \left(\prod_j \sum_{k_j=1}^{\kappa_j} \prod_i \frac{(m_{ij}p_i)^{b_{k_j i}^{(j)}}}{b_{k_j i}^{(j)}!} \right) \times \prod_i p_i^{\delta-1} \times \\ \prod_i \frac{\Gamma(\gamma_i(C\epsilon+1))}{(\Gamma(\gamma_i\epsilon))^{C-1}\Gamma(\gamma_i(\epsilon+1))} \prod_j (m_{ij})^{t_{ij}+\gamma_i(\epsilon+1(i=j))-1} \\ \propto \sum_{k_1=1}^{n_1} \cdots \sum_{k_C=1}^{n_C} \left(\prod_i \frac{\Gamma(\gamma_i(C\epsilon+1)) p_i^{\sum_j b_{k_j i}^{(j)}-1}}{(\Gamma(\gamma_i\epsilon))^{C-1}\Gamma(\gamma_i(\epsilon+1))} \times \right. \\ \left. \prod_j \frac{(m_{ij})^{b_{k_j i}^{(j)}+t_{ij}+\gamma_i(\epsilon+1(i=j))-1}}{b_{k_j i}^{(j)}!} \right)$$

Given $k_1, \ldots, k_C$ and i, the product $\prod_{j=1}^{C} (m_{ij})^{b_{k_j i}^{(j)}+t_{ij}+\gamma_i(\epsilon+1(i=j))-1}$ is the kernel of a $Dirichlet(b_{k_1 i}^{(1)} + t_{i1} + \gamma_i\epsilon, \ldots, b_{k_i i}^{(i)} + t_{ii} + \gamma_i(\epsilon+1), \ldots, b_{k_C i}^{(C)} + t_{iC} + \gamma_i\epsilon)$ distribution. Hence, integrating $\mathbf{M}$ out

with respect to the order $\prod_{i=1}^{C}\prod_{j=1}^{C} dm_{ij}$, we are left with

$$p(\mathbf{p} \mid \mathbf{v}, \mathbf{T}, \boldsymbol{\gamma}) \propto \sum_{k_1=1}^{n_1} \cdots \sum_{k_C=1}^{n_C} w_{k_1,k_2,\ldots,k_C}(\boldsymbol{\gamma}, \epsilon) \prod_i p_i^{\sum_j b_{k_j i}^{(j)}+\delta-1}$$

where $w_{k_1,k_2,\ldots,k_C}(\boldsymbol{\gamma}, \epsilon) = \prod_i \frac{\Gamma(\gamma_i(C\epsilon+1)) \prod_{j=1}^{C} \Gamma(b_{k_j i}^{(j)}+t_{ij}+\gamma_i(\epsilon+1(i=j)))}{(\Gamma(\gamma_i\epsilon))^{C-1}\Gamma(\gamma_i(\epsilon+1))\Gamma(\sum_j(b_{k_j i}^{(j)}+t_{ij})+\gamma_i(C\epsilon+1))\prod_j b_{k_j i}^{(j)}!}$. Hence,

$$\mathbf{p} \mid \mathbf{v}, \mathbf{T} \sim \sum_{k_1=1}^{n_1} \cdots \sum_{k_C=1}^{n_C} \left(\left(\int \frac{1}{W(\boldsymbol{\gamma}, \epsilon)} w_{k_1,k_2,\ldots,k_C}(\boldsymbol{\gamma}, \epsilon) dF(\boldsymbol{\gamma})\right) \times \right.$$
$$\left. Dirichlet(\sum_j b_{k_j 1}^{(j)} + \delta, \ldots, \sum_j b_{k_j C}^{(j)} + \delta)\right)$$

where $W(\boldsymbol{\gamma}, \epsilon) = \sum_{k_1=1}^{n_1} \cdots \sum_{k_C=1}^{n_C} w_{k_1,k_2,\ldots,k_C}(\boldsymbol{\gamma}, \epsilon)$. Without loss of generality, let the first row of each $\mathbf{B}^{(j)}$ represent the partition of v_j which allocates v_j to the jth component and 0 to all the other components. For any $(k_1, k_2, \ldots, k_C)' \neq \mathbf{1}_C$, we have

$$\lim_{\epsilon \to 0} \frac{w_{k_1,k_2,\ldots,k_C}(\boldsymbol{\gamma}, \epsilon)}{w_{1,1,\ldots,1}(\boldsymbol{\gamma}, \epsilon)} = \prod_i \left(\frac{\Gamma(\sum_j t_{ij} + v_i + \gamma_i)\Gamma(b_{k_i i}^{(i)} + t_{ii} + \gamma_i)}{\Gamma(\sum_j(b_{k_j i}^{(j)} + t_{ij}) + \gamma_i)\Gamma(v_i + t_{ii} + \gamma_i)} \times \right.$$
$$\left. \left(\prod_{j \neq i} \lim_{\epsilon \to 0} \frac{\Gamma(b_{k_j i}^{(j)} + t_{ij} + \gamma_i\epsilon)}{\Gamma(t_{ij} + \gamma_i\epsilon)} \right) \right)$$

If $b_{k_j i}^{(j)} = 0$, the ratio $\frac{\Gamma(b_{k_j i}^{(j)}+t_{ij}+\gamma_i\epsilon)}{\Gamma(t_{ij}+\gamma_i\epsilon)}$ is one. However, since $(k_1, k_2, \ldots, k_C)' \neq \mathbf{1}_C$, we have atleast one pair $i \neq j$ such that $b_{k_j i}^{(j)} \geqslant 1$ and consequently

$$\frac{\Gamma(b_{k_j i}^{(j)} + t_{ij} + \gamma_i\epsilon)}{\Gamma(t_{ij} + \gamma_i\epsilon)} = \prod_{s=0}^{b_{k_j i}^{(j)}-1} (s + t_{ij} + \gamma_i\epsilon) \xrightarrow{\epsilon \to 0} 0$$

since T is diagonal. Hence, $w_{1,1,\ldots,1}$ dominates all the other weights in the limiting case. Since each of the scaled weights are less than one, using dominated convergence theorem,

$$\lim_{\epsilon \to 0} \int \frac{1}{W(\boldsymbol{\gamma}, \epsilon)} w_{k_1,k_2,\ldots,k_C}(\boldsymbol{\gamma}, \epsilon) dF(\boldsymbol{\gamma}) = 1((k_1, k_2, \ldots, k_C)' = \mathbf{1})$$

and hence $\lim_{\epsilon \to 0} p(\mathbf{p} \mid \mathbf{v}, \mathbf{T}) \propto \prod_i p_i^{\sum_j b_{1i}^{(j)}+\delta-1} = \prod_i p_i^{v_i+\delta-1}$. □

Theorem 3.1. We proof only for the case $K = 2$ as the same proof generalizes for arbitrary K. We simplify the notation for the proof. Let v_{st} denote the number of instances in $\mathcal{U}$ assigned to

class s by algorithm 1, and class t by algorithm 2. We write $\mathbf{M}^{(1)} = \mathbf{M}$, $\mathbf{M}^{(2)} = \mathbf{N}$, $\mathbf{T}^{(1)} = \mathbf{T}$ and $\mathbf{T}^{(2)} = \mathbf{U}$ to get rid of the superscripts. Also, let $\mathbf{B}_{(st)} = (b_{li}^{(st)})$ denote a $\kappa_{st} \times C$ matrix formed by stacking row-wise all possible partitions of v_{st} into C non-negative integers. Here $\kappa_{st} = \binom{v_{st}+C-1}{C-1}$ denotes the total number of such partitions. Let $\mathbf{h} = (h_{11}, h_{12}, \ldots, h_{CC})'$ denote a generic index vector such that each $h_{st} \in \{1, 2, \ldots, \kappa_{st}\}$ indexes a partition of v_{st} and $\mathcal{H}$ denote the collection of all such $\mathbf{h}$'s. Then likelihood for $(\mathbf{a}_1, \mathbf{a}_2, \ldots, \mathbf{a}_N)'$ is

$$\begin{aligned}\prod_{s=1}^{C}\prod_{t=1}^{C}\left(\sum_{l=1}^{\kappa_{st}}\prod_{i=1}^{C}\frac{(p_i m_{is} n_{it})^{b_{li}^{(st)}}}{b_{li}^{(st)}!}\right) &= \sum_{\mathbf{h}\in\mathcal{H}}\prod_{i=1}^{C}\prod_{s=1}^{C}\prod_{t=1}^{C}\frac{(p_i m_{is} n_{it})^{b_{h_{st}i}^{(st)}}}{b_{h_{st}i}^{(st)}!}\\
&= \sum_{\mathbf{h}\in\mathcal{H}}\left(\frac{1}{c_{\mathbf{h}}}\prod_{i=1}^{C} p_i^{\sum_{s=1}^{C}\sum_{t=1}^{C} b_{h_{st}i}^{(st)}}\times\right.\\
&\qquad\left.\prod_{s=1}^{C} m_{is}^{\sum_{t=1}^{C} b_{h_{st}i}^{(st)}}\prod_{t=1}^{C} n_{it}^{\sum_{s=1}^{C} b_{h_{st}i}^{(st)}}\right)\end{aligned}$$

where $c_{\mathbf{h}}$ is a constant term free of the parameters.

Incorporating the priors and marginalizing with respect to $\mathbf{M}$ and $\mathbf{N}$ we have

$$\begin{aligned}p(\mathbf{p}\mid\mathbf{v}^*, \mathbf{T}, \mathbf{U}, \boldsymbol{\gamma}^{(1)}, \boldsymbol{\gamma}^{(2)}) \propto &\sum_{\mathbf{h}\in\mathcal{H}}\frac{1}{c_{\mathbf{h}}}\prod_{i=1}^{C}\left(p_i^{\sum_{s=1}^{C}\sum_{t=1}^{C} b_{h_{st}i}^{(st)}+\delta-1}\times\right.\\
&\frac{\prod_{s=1}^{C}\Gamma(\sum_{t=1}^{C} b_{h_{st}i}^{(st)} + t_{is} + \gamma_i^{(1)}(\epsilon + I(i=s))}{\Gamma\left(\sum_{s=1}^{C}\left(\sum_{t=1}^{C} b_{h_{st}i}^{(st)} + t_{is}\right) + \gamma_i^{(1)}(C\epsilon+1)\right)}\times\\
&\left.\frac{\prod_{t=1}^{C}\Gamma(\sum_{s=1}^{C} b_{h_{st}i}^{(st)} + u_{it} + \gamma_i^{(2)}(\epsilon + I(i=t))}{\Gamma\left(\sum_{t=1}^{C}\left(\sum_{s=1}^{C} b_{h_{st}i}^{(st)} + u_{it}\right) + \gamma_i^{(2)}(C\epsilon+1)\right)}\right)\\
\propto &\sum_{\mathbf{h}\in\mathcal{H}} w_{\mathbf{h}}(\gamma^{(1)}, \boldsymbol{\gamma}^{(2)}, \epsilon)\prod_{i=1}^{C} p_i^{\sum_{s=1}^{C}\sum_{t=1}^{C} b_{h_{st}i}^{(st)}+\delta-1}\end{aligned}$$

where $w_{\mathbf{h}}(\gamma^{(1)}, \boldsymbol{\gamma}^{(2)}, \epsilon)$ is the weight comprising of all the terms not involving p_i's. Now, let $\mathcal{H}^*$ denote the subset of $\mathcal{H}$ such that for all $\mathbf{h}^* = (h_{11}^*, h_{12}^*, \ldots, h_{CC}^*)' \in \mathcal{H}^*$, each index h_{st}^* corresponds to a partition of v_{st} which allocates v_{st} to the sth partition and zero to all the other partitions. Clearly, for any $\mathbf{h}^*$, $\sum_{s=1}^{C}\sum_{t=1}^{C} b_{h_{st}^*i}^{(st)} = \sum_{s=1}^{C}\sum_{t=1}^{C} v_{st} I(i=s) = \sum_{s=1}^{C} I(i=s) v_s = v_i$.

Let ζ denote a generic positive constant which does not depend on ϵ. We absorb terms of the form $\lim_{\epsilon\to 0}\Gamma(x + \mathcal{O}(\epsilon))$ where x is always greater than 1 into ζ, as these limits will be non-zero.

Noting that $t_{is} = 0$ if $s \neq i$, for any $\mathbf{h}^* \in \mathcal{H}$ and $\mathbf{h} \in \mathcal{H} \setminus \mathcal{H}^*$, we have

$$\lim_{\epsilon \to 0} \frac{w_{\mathbf{h}}}{w^*_{\mathbf{h}}} = \zeta \prod_{i=1}^{C} \frac{\prod_{s \neq i} \Gamma(\sum_{t=1}^{C} b^{(st)}_{h_{st}i} + \gamma_i^{(1)}\epsilon)}{\prod_{s \neq i} \Gamma(\gamma_i^{(1)}\epsilon)} \frac{\prod_{t \neq i} \Gamma(\sum_{s=1}^{C} b^{(st)}_{h_{st}i} + u_{it} + \gamma_i^{(2)}\epsilon)}{\prod_{t \neq i} \Gamma(\sum_{s=1}^{C} b^{(st)}_{h^*_{st}i} + u_{it} + \gamma_i^{(2)}\epsilon)}$$

Since u_{it}'s are greater than zero and there exists at least one pair (i, s) such that $\sum_{t=1}^{C} b^{(st)}_{h_{st}i} > 0$, the result follows. □

S5. Detailed analysis of the simulation results

In this Section we present a much more thorough analysis of the simulation study, as well as investigate additional methods to generate population-level class probabilities in the target-domain.

S5.1 *Impact of difference in marginal class distributions between source- and target-domains*

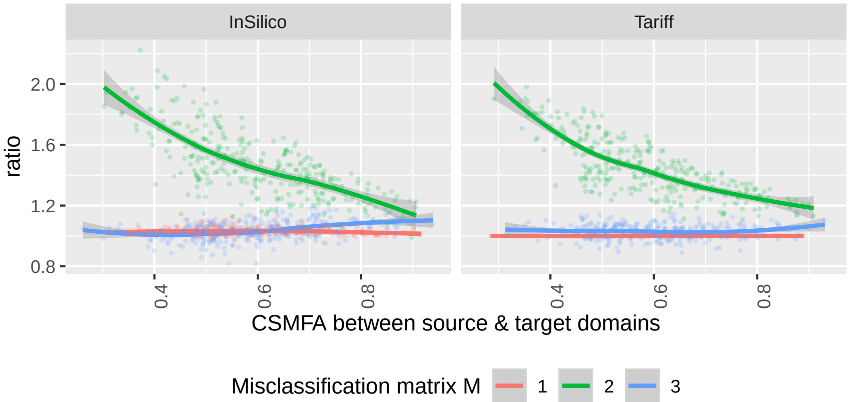

Fig. S1. Ratio of CSMFA of baseline model and transfer learner

We first investigate how the performance of our Bayesian transfer learning model is impacted by the disparity in class distribution between source- and target-domains. Figure S1 plots the smoothed ratio of the CSMFA of the baseline estimates and their calibrated analogs from our model, as a function of the true CSMFA between the class probabilities $\mathbf{p}_{\mathcal{G}}$ and $\mathbf{p}_{\mathcal{U}}$ in the source- and target-domains. The left panels correspond to data generated using InSilicoVA

and hence assesses the performance of InSilicoVA$_{\mathcal{G}}$ and InSilicoVA$_{BTL}$ by plotting the ratio CSMFA(InSilicoVA$_{BTL}$)/ CSMFA(InSilicoVA$_{\mathcal{G}}$). Similarly, the right panels correspond to data generated using Tariff and compares the estimates from Tariff$_{\mathcal{G}}$ and Tariff$_{BTL}$. We only present the results for $n = 400$, as we will discuss the role of n in Section S5.3.

We first note that when data was generated using the misclassification matrix $\mathbf{M}_1 = \mathbf{I}$, the ratio is exactly one. This corroborates the result in Theorem 2.1 that if A classifies flawlessly in the target-domain, then the baseline and transfer learning estimates are same. For $\mathbf{M}_3$, i.e. when the misclassification rate is small, the ratio is also close to one with the transfer learning estimate being slightly more accurate in general. For $\mathbf{M}_2$, which portrays the scenario where the the baseline learner trained on source- domain is systematically and substantially biased, one can clearly see the benefit of transfer learning. The CSMFA is significantly better after transfer learning. It also nicely shows the utility of transfer learning as a function of $x = CSMFA(\mathbf{p}_{\mathcal{G}}, \mathbf{p}_{\mathcal{U}})$ (on the x-axis). Unsurprisingly, the ratio is decreasing with increasing x. When x is small, i.e., there exists much disparity in the marginal class distributions between the source- and target-domains, the ratio is close to two, implying that transfer learning yields near 100% gain in accuracy. When x is close to one, the improvement is much less stark, which is expected as in this scenario the class probabilities in the non-local and local populations are almost identical.

S5.2 *Biases in estimates of probabilities for each class*

We also look at the biases in the estimates of each of the four class probabilities in Figure S2. The top and bottom rows correspond to data generated using InSilicoVA and Tariff respectively. The three columns correspond to three choices of $\mathbf{M}$. We see that there is almost no bias for $\mathbf{M}_1$ for all the methods, for $\mathbf{M}_3$ the baseline Tariff$_{\mathcal{G}}$ estimates are generally unbiased, whereas the baseline InSilicoVA$_{\mathcal{G}}$ show small biases. However, for $\mathbf{M}_2$ we see the substantial biases in the estimates from both the baseline approaches. As expected due to the specification of $\mathbf{M}_2$,

the baseline learners underestimate $P(Diarrhea/Dysentry)$ (Cause 2) and $P(Sepsis)$ (Cause 3) while overestimating $P(Pneumonia)$ (Cause 1) and $P(Other)$ (Cause 4) are overestimated. The transfer learning estimates Tariff$_{BTL}$ and InSilicoVA$_{BTL}$ are unbiased for all the settings.

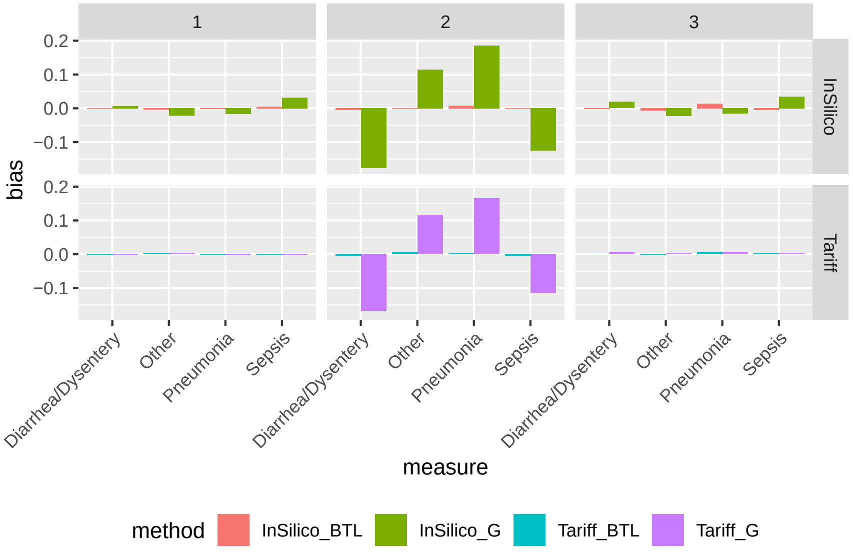

Fig. S2. Biases in the average estimates of individual cause prevalences

S5.3 *Role of limited labeled data in target-domain*

We now investigate the role of the sample size n and the marginal class distribution $\mathbf{p}_{\mathcal{L}}$ of $\mathcal{L}$. Additionally, as an alternate to our transfer learning approach, we also consider including the local labeled data $\mathcal{L}$ as part of the training data for the CCVA algorithms. So, we have four more methods Tariff$_{\mathcal{L}}$, Tariff$_{\mathcal{G}\cup\mathcal{L}}$, InSilicoVA$_{\mathcal{L}}$ and InSilicoVA$_{\mathcal{G}\cup\mathcal{L}}$, where the sub-scripts indicate the training data used.

When data is generated using InSilicoVA, Figure S3 provides the boxplots of CSMF accuracy of the methods for all the scenarios as a function of n (rows), choice of $\mathbf{M}$ (columns) and ρ — the CSMFA-range between $\mathbf{p}_{\mathcal{L}}$ and $\mathbf{p}_{\mathcal{U}}$ (x-axis in each sub-figure).

We unpack many different conclusions from this Figure. First we look at the performances

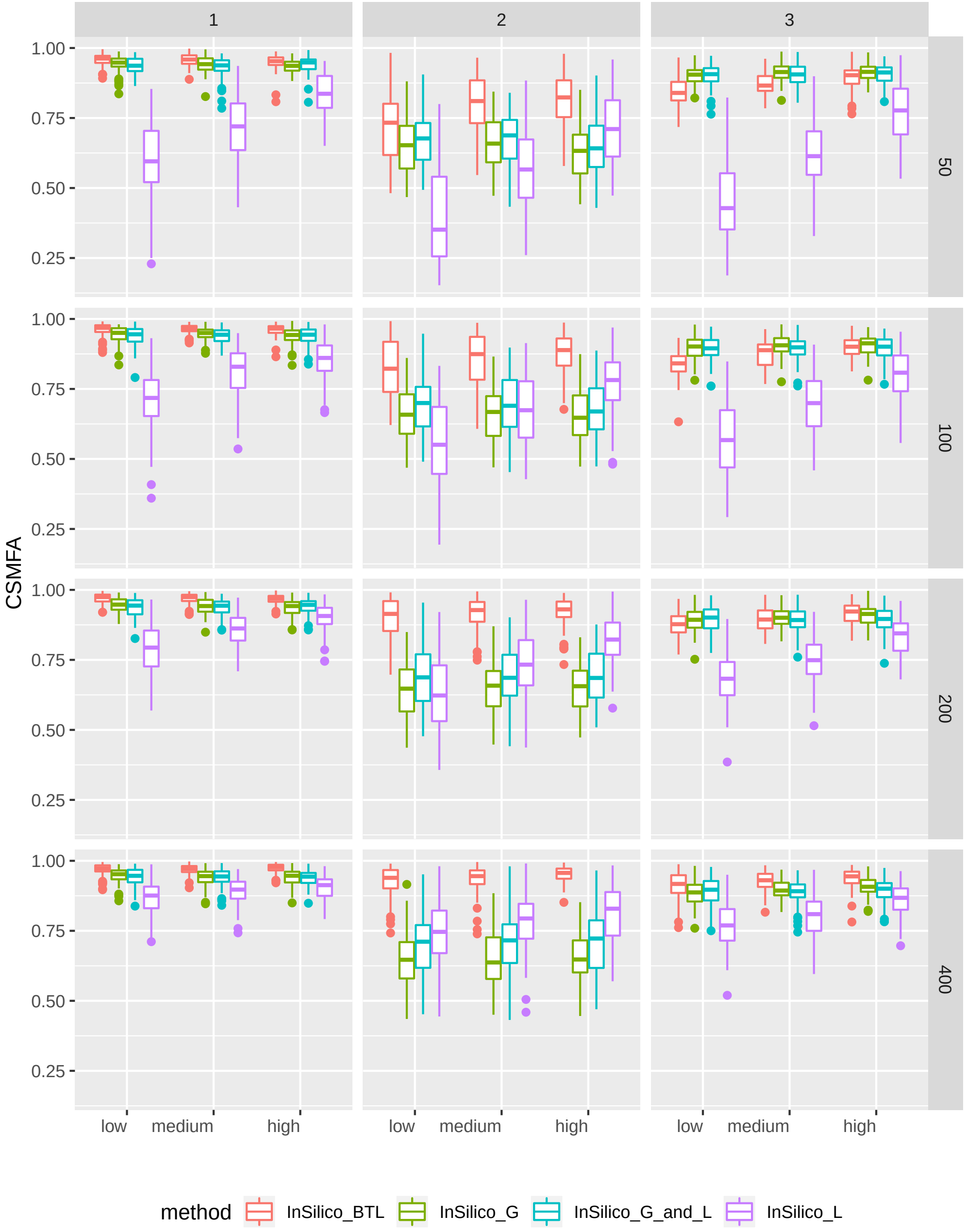

Fig. S3. CSMFA for four InSilicoVA based methods for data generated using InSilicoVA

of InSilicoVA$_{\mathcal{G}}$ and InSilicoVA$_{BTL}$. These two methods was already compared in Section S5.1, but only for fixed $n = 400$ and averaged across all ρ. Here, further analyzing the performances as a function of n and ρ, we see that the CSMFA of calibrated VA using our model increases with increase in n. Also, there is a drastic gain in precision of the calibrated estimates with the confidence bands shortening with increase in n from 50 to 400. Additionally, we see that the CSMFA for InSilicoVA$_{BTL}$ increases as ρ goes from *low* to *medium* to *high*, although the gain is not as drastic. This indicates that the transfer learning procedure, while being reasonably robust to the value of ρ, does benefit to a small extent from improved concordance between the class probabilities in $\mathcal{L}$ and $\mathcal{U}$. Of course, InSilicoVA$_{\mathcal{G}}$ is not affected by either n or ρ. In general, for $\mathbf{M}_3$, we see that only when both n is small and ρ is *low*, the InSilicoVA$_{\mathcal{G}}$ produces slightly better estimates than InSilicoVA$_{BTL}$. For all other cases, InSilicoVA$_{BTL}$ yields higher or similar CSMF. For $\mathbf{M}_2$, we see InSilicoVA$_{BTL}$ dominates InSilicoVA$_{\mathcal{G}}$ across all scenarios. The gains from increase in n and ρ are evident here as well. Finally, for $\mathbf{M}_1$, the performance of InSilicoVA$_{BTL}$ is identical to InSilicoVA$_{\mathcal{G}}$, as is guaranteed by Theorem 2.1, and is not affected by n or ρ.

Next, we look at the performance of InSilicoVA$_{\mathcal{L}}$ and InSilicoVA$_{\mathcal{G}\cup\mathcal{L}}$. For $\mathbf{M}_1$ and $\mathbf{M}_3$, InSilicoVA$_{\mathcal{L}}$ performs quite poorly, generally producing the lowest CSMF. InSilicoVA$_{\mathcal{L}}$ is also highly sensitive to both ρ and n, yielding highly variable and inaccurate estimates for *low* ρ and n, and improving sharply as either increases. Only for $\mathbf{M}_2$, for large n or large ρ, it does better than InSilicoVA$_{\mathcal{G}}$. As this setting portrays substantial difference in the conditional distributions between the source- and target population, InSilicoVA$_{\mathcal{L}}$, trained on local data, does better. CSMFA from InSilicoVA$_{\mathcal{G}\cup\mathcal{L}}$, which uses both the source- and target labeled data in the training, generally lies between the CSMFA from InSilicoVA$_{\mathcal{G}}$ and InSilicoVA$_{\mathcal{L}}$, and is much closer to the former as $\mathcal{G}$ far outnumbers $\mathcal{L}$. Finally, comparing InSilicoVA$_{\mathcal{L}}$ and InSilicoVA$_{\mathcal{G}\cup\mathcal{L}}$ to InSilicoVA$_{BTL}$, we see that the InSilicoVA$_{BTL}$ does substantially better than InSilicoVA$_{\mathcal{L}}$ uniformly across the scenarios, and than InSilicoVA$_{\mathcal{G}\cup\mathcal{L}}$ across all scenarios except when both n

is small and ρ is *low*. This shows that with a small labeled dataset in the target-domain, our transfer learning approach is a more resourceful way of exploiting this limited data and results in more accurate and robust estimates. The analogous results for data generated using Tariff, provided in Figure S10 of the supplement, reveals similar trends.

S5.4 *Comparison with the naive transfer learning*

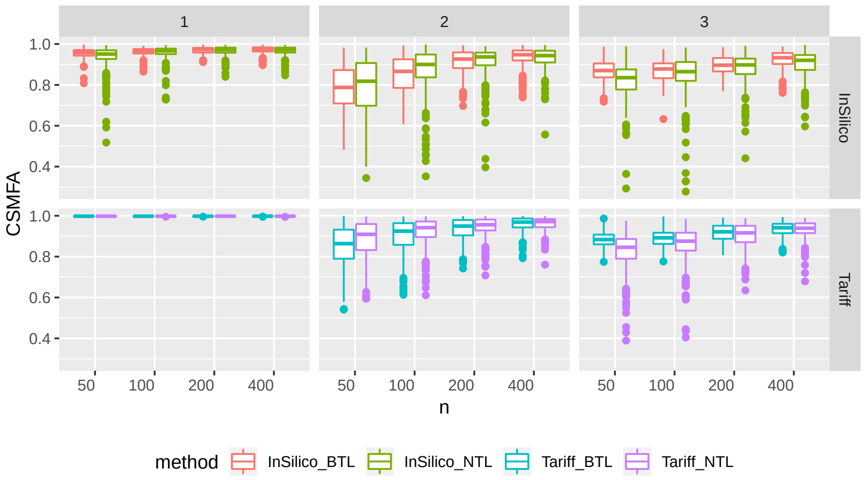

Fig. S4. CSMFA of naive and Bayesian transfer learning

To understand the importance of the Bayesian shrinkage or regularization used in the the transfer learning, we also compare with the naive transfer learning based on MLE, outlined in Section 2. We refer to the naive transfer learning using Tariff and InSilicoVA respectively as Tariff_{NTL} and InSilicoVA_{NTL}. Figure S4 compares the CMSFA for the naive and Bayesian regularized transfer learning approaches. Once again, the top and bottom row corresponds to data generated using Tariff and InSilicoVA respectively, the three columns are for three choices of $\mathbf{M}$ and within each setting, we plot the boxplots of CSMFA as a function of n.

We see that, generally the median estimates from the naive approach is similar to the ones produced using the Bayesian regularized analog. However, there is notable difference in the variability

of CSMFA, with the naive approach producing a wide range of values with several extreme estimates. The problem is exacerbated for smaller values of n. The results from the Bayesian model are more stable with uniformly lesser variation across all the settings. It is evident, that in real data analysis, where the truth is unknown, the Bayesian model will be much more reliable than the MLE based solution which seems to be quite likely to yield absurd estimates.

S5.5 *Performance of ensemble models*

We now analyze the performance of the joint (Ensemble$_J$) and independent (Ensemble$_I$) ensemble transfer learning models introduced in Section 3. These models use output from both Tariff and InSilicoVA whereas the single-classifier models Tariff$_{BTL}$ and InSilicoVA$_{BTL}$ only use the output from one CCVA algorithm. For a given dataset, we define

$$\delta = \max(\text{CSMFA}(\text{InSilicoVA}_{BTL}), \text{CSMFA}(\text{Tariff}_{BTL})) - \min(\text{CSMFA}(\text{InSilicoVA}_{BTL}), \text{CSMFA}(\text{Tariff}_{BTL})).$$

In other words, δ denotes the difference in CSMFA of the calibrated VA using the most and least accurate classifiers. A small δ implies transfer learning with either of the baseline classifiers yield similar results, whereas larger values of δ clearly insinuate that transfer learning with one of the baseline classifiers is more accurate than the other one. For an ensemble method that aims to guard against inclusion of an inaccurate method, one would expect that CSMFA for the ensemble method should be closer to that of the best performing method. Equivalently, if

$$\nu = CSMFA(Ensemble) - \min(\text{CSMFA}(\text{InSilicoVA}_{BTL}), \text{CSMFA}(\text{Tariff}_{BTL})),$$

where Ensemble refers to either Ensemble$_I$ or Ensemble$_J$, then ν should be greater than $\delta/2$.

Figure S5 plots ν as a smoothed function of δ. We first note that, for $\mathbf{M}_1$ (red lines), the (ν, δ) curve for the joint sampler nearly coincides with the 45-degree line. Since, in our data generation process, under $\mathbf{M}_1$ one of the classifiers is fully accurate, this empirically verifies the

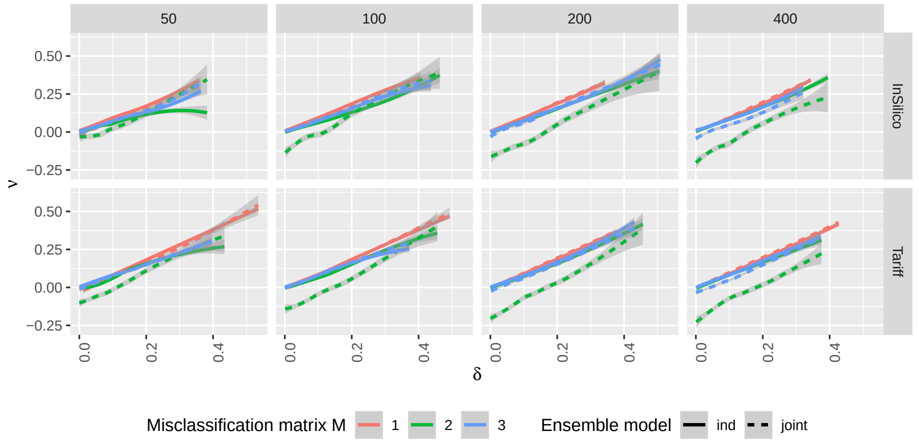

Fig. S5. Performance of the ensemble models

theoretical guarantee in Theorem 3.1, that in such settings posterior mean of class probabilities from the ensemble approach is same as that from the best classifier. While the independent ensemble model does not enjoy this theoretical property, in practice we see that for $\mathbf{M}_1$, ν is also identical to δ. For $\mathbf{M}_2$ and $\mathbf{M}_3$, across all scenarios, ν is close to δ, i.e. estimates from both the Ensemble$_J$ and Ensemble$_I$ models generally aligns much closer to the best performing single-classifer transfer learner. There are no significant trends with respect to either the size of $\mathcal{L}$ (n) or the data generating algorithm – InSilicoVA (top-row) and Tariff (bottom-row). The Ensemble$_I$ model seems to do slightly better than the joint model. Since, it is also the faster model, we only use this version of the ensemble model for subsequent analysis. The performance of the ensemble samplers is quite reassuring especially for larger δ, as it demonstrates the robustness to inclusion of a bad method via averaging over multiple methods. As ν seems to be substantially greater than $\delta/2$ for most of the curves, it also shows why our model based method averaging is superior to simply taking average of the estimated class-probabilities from the different methods, which is much more affected by the worst method.

S5.6 *Informative shrinkage*

If we have prior knowledge on $\mathbf{M}$, we can use this for informative shrinkage, rather than shrinking towards the source- predictor. For example, when the true matrix is $\mathbf{M}_2$, if we assume that it was known apriori that label 2 is often misclassified as label 1, and label 3 is often misclassified as label 4, then instead of shrinking $\mathbf{M}$ towards the identity matrix, we can shrink $\mathbf{M}$ towards transition matrices of the form

$$\begin{pmatrix} 1 & 0 & 0 & 0 \\ m_{21} & m_{22} & 0 & 0 \\ 0 & 0 & m_{33} & m_{34} \\ 0 & 0 & 0 & 1 \end{pmatrix}.$$

To do this *informative shrinkage*, we can let

$$\mathbf{X} = \begin{bmatrix} 1 & 0 & 0 & 0 \\ 1 & 1 & 0 & 0 \\ 0 & 0 & 1 & 1 \\ 0 & 0 & 0 & 1 \end{bmatrix}$$

and use an informed prior on $\mathbf{M}$ such that

$$\mathbf{M}_{i*} \overset{ind}{\sim} \text{Dirichlet}(\gamma_i(\mathbf{X}_{i*} + \epsilon\mathbf{1})), i = 1, 2, 3, 4$$

This prior would reflect our knowledge of which causes are likely to be misclassified by the algorithm. We then modify our Gibbs updates as follows:

$$\mathbf{M}_{i*} \mid \cdot \sim Dirichlet(\mathbf{B}_{i*} + \mathbf{T}_{i*} + \lambda_i(\mathbf{X}_{i*} + \epsilon\mathbf{1}))$$
$$p(\gamma_i \mid \cdot) \propto \frac{\Gamma(C\gamma_i\epsilon + \gamma_i \cdot \sum_j \mathbf{1}(\mathbf{X}_{ij} = 1))}{\prod_j \Gamma(\gamma_i\epsilon + \gamma_i\mathbf{1}(\mathbf{X}_{ij} = 1))} \gamma_i^{\alpha-1} \exp(-\beta\gamma_i) \prod_j m_{ij}^{\gamma_i\epsilon + \gamma_i\mathbf{1}(\mathbf{X}_{ij}=1)}$$

While the choice of prior is less likely to affect the results with a larger calibration set size, we can compare the CSMFA when using a smaller calibration set size in Figure S6 below.

We see that with a sample size of 50 for our $\mathcal{L}$, using the informed prior on $\mathbf{M}$ leads to improved CSMFA. When the sample size for $\mathcal{L}$ grows to 100, there is still some improvement in CSMFA

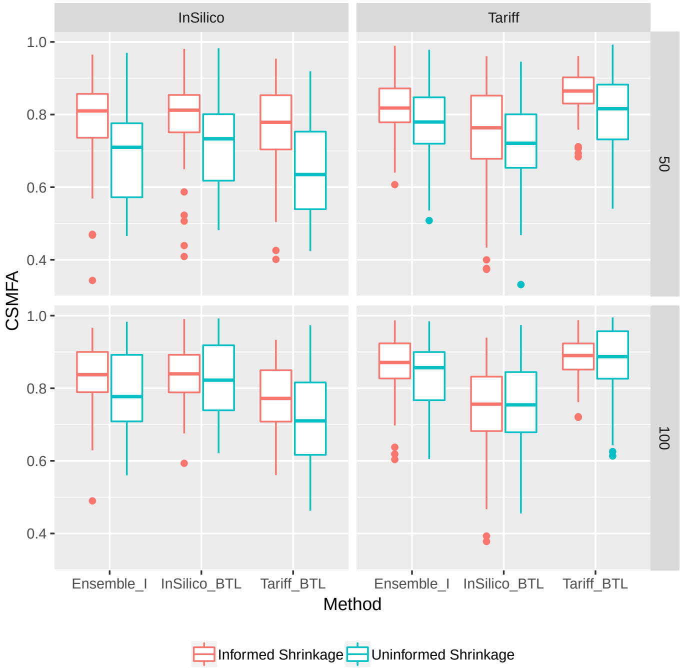

Fig. S6. Comparison between informative and non-informative (default) shrinkage.

with informed shrinkage when the data is generated using InSilicoVA, and the performance is nearly identical between the two models when the data is generated by Tariff.

S5.7 *Individual level classification*

As mentioned earlier, predicting individual classes is not our primary goal. Nonetheless, we have outlined a simple way to obtain individual predictions using our transfer learning model. Here we compared its accuracy using the Chance Corrected Concordance (Murray *and others*, 2011) defined as

$$\text{CCC} = \frac{1}{C}\sum_{i=1}^{C}\frac{\frac{TP_i}{TP_i+TN_i} - \frac{1}{N}}{1-\frac{1}{N}}$$

where TP_i and TN_i denote the true positive and true negative rates for class i. We only analyze the case when when the data is generated using InSilicoVA (Figure S7). The roles are

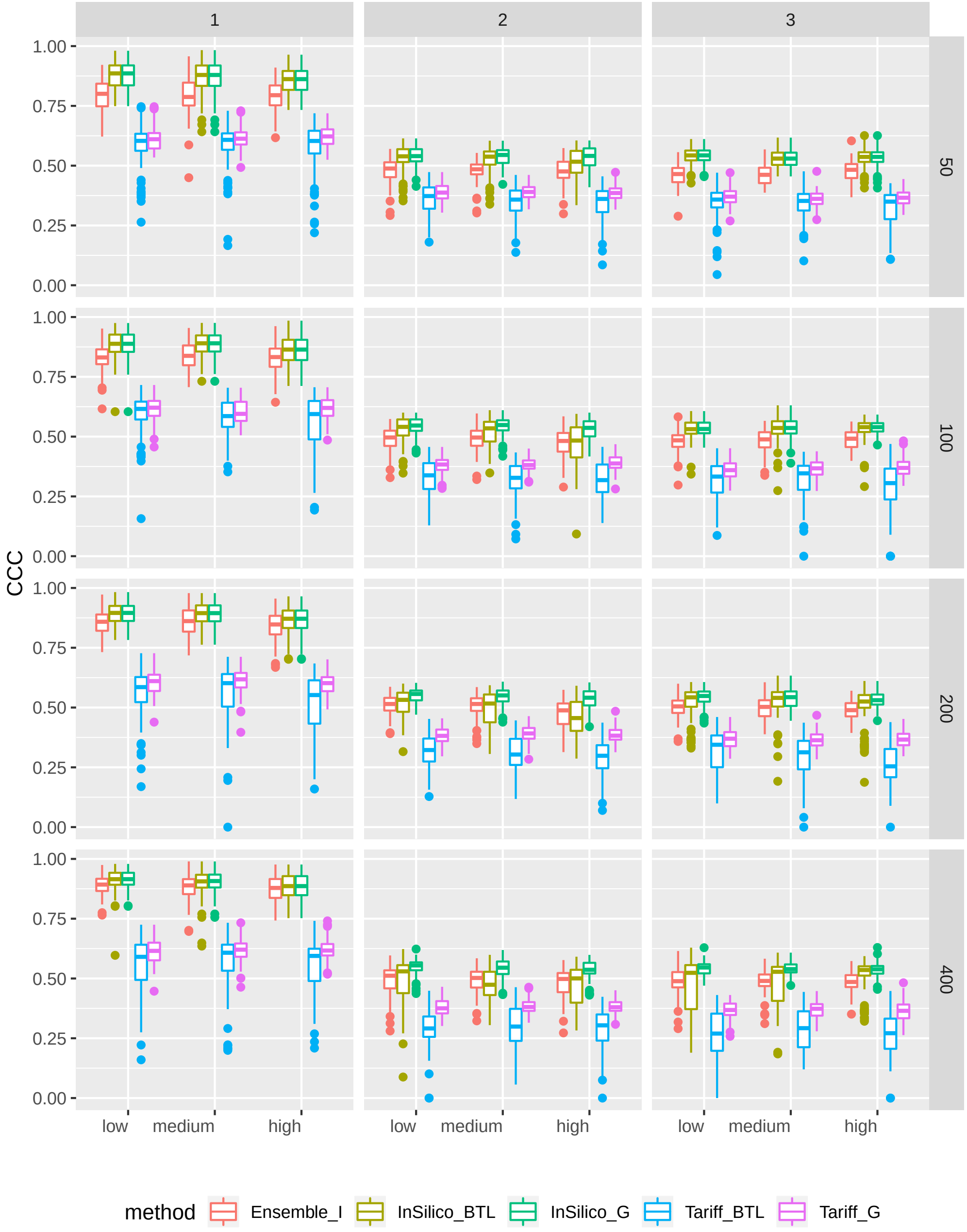

Fig. S7. CCC when data is generated using InSilicoVA

simply reversed when data is generated using Tariff (Figure S11). We see in Figure S7 that CCC for InSilicoVA$_{\mathcal{G}}$ and InSilicoVA$_{BTL}$ are better than those of Tariff$_{\mathcal{G}}$ and Tariff$_{BTL}$ respectively. This is expected as analyzing data using the true model is expected to perform better than the misspecified model. CCC from the transfer learning (InSilicoVA$_{BTL}$) and baseline (InSilicoVA$_{\mathcal{G}}$) versions of the same CCVA algorithm, which was used in data generation, were similar. For the misspecified model, the baseline Tariff$_{\mathcal{G}}$ produced slightly better CCC than the transfer learning Tariff$_{BTL}$, although this improvement in performance is minor. Overall, these results indicate that if individual prediction is of interest, then perhaps more advanced methods need to be considered than the simple approach we have outlined. However, even using our crude approach, we see that the ensemble model (Ensemble$_I$) produces CCC closer to InSilicoVA$_{BTL}$ and InSilicoVA$_{\mathcal{G}}$, and much better than the CCC obtained by both Tariff$_{BTL}$ and Tariff$_{\mathcal{G}}$. This once again furnishes evidence of the robust performance of the ensemble model, and in practice, when we will not know which algorithm works best, using the ensemble model will safeguard against choosing a bad algorithm.

S6. Comparing marginal symptom distributions between $\mathcal{L}$ and $\mathcal{U}$

To show that our method also does not assume similar symptom marginal distributions between $\mathcal{L}$ and $\mathcal{U}$, in Figure S8 we plot the proportion of presence ("Yes") of each symptom in $\mathcal{U}$ and $\mathcal{L}$ (for 10 randomly selected samples of $\mathcal{L}$) in India and Tanzania for this analysis. We see that while many symptoms are rare in both $\mathcal{L}$ and $\mathcal{U}$ (clustering near (0,0)), the marginal distributions of the symptoms do not have to match between $\mathcal{U}$ and $\mathcal{L}$, with the symptom proportion in $\mathcal{L}$ varying considerably on both sides (up to $\pm15\%$) than the analogous quantity in $\mathcal{U}$)

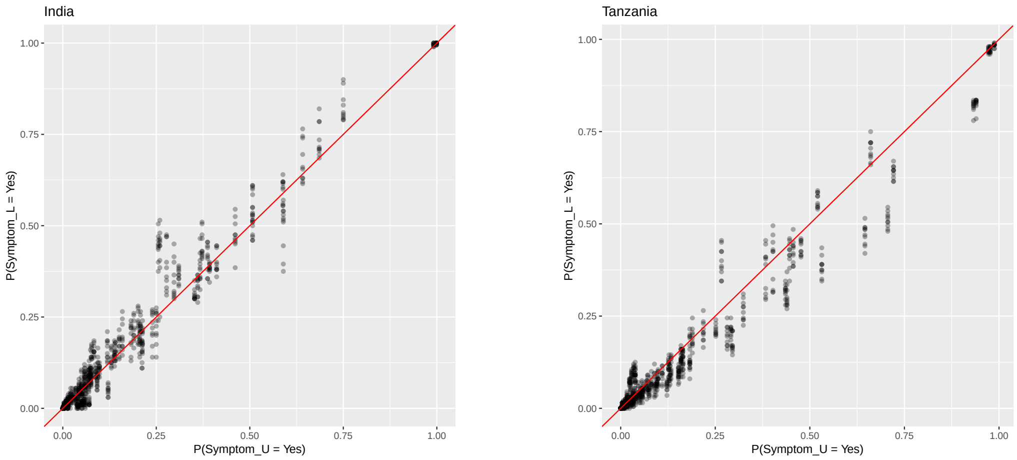

Fig. S8. Scatterplot of the symptom proportions in $\mathcal{U}$ and 10 randomly sampled choices of $\mathcal{L}$. The red line is the $x = y$ line.

S7. Impact of number of cause categories for PHMRC analysis

To investigate the effect of adding more causes of death on the transfer learning CSMFA, we added "Malaria" and "Sepsis", which were part of the "Other Infectious" category, as individual causes. Due to the nature of the CSMFA metric, it is difficult to directly compare accuracy on estimating a probability vector of length 5 versus a probability vector of length 7. Hence, after getting the transfer learning CSMF estimates for the 7 cause categories, we aggregated the 7 cause CSMFs back to the orignal 5 cause CSMFs, i.e., we added the CSMF estimates for "Malaria" and "Sepsis" to the CSMF estimate for "Other Infectious", so that we could fairly compare the CSMFA when using 5 versus 7 causes.

Looking at Figure S9, we see that there is actually very little change in the CSMFA when using individual algorithms. This would indicate that when we only are using one algorithm, the additional causes are not causing substantial shrinkage in the estimates of $\mathbf{M}$. We would expect that as the number of causes grows even larger and the size of $\mathcal{L}$ is small, there are fewer number of samples per cause category leading to more shrinkage towards the source- predictor and hence worse performance. We only see this for the ensemble model and for sample sizes 50

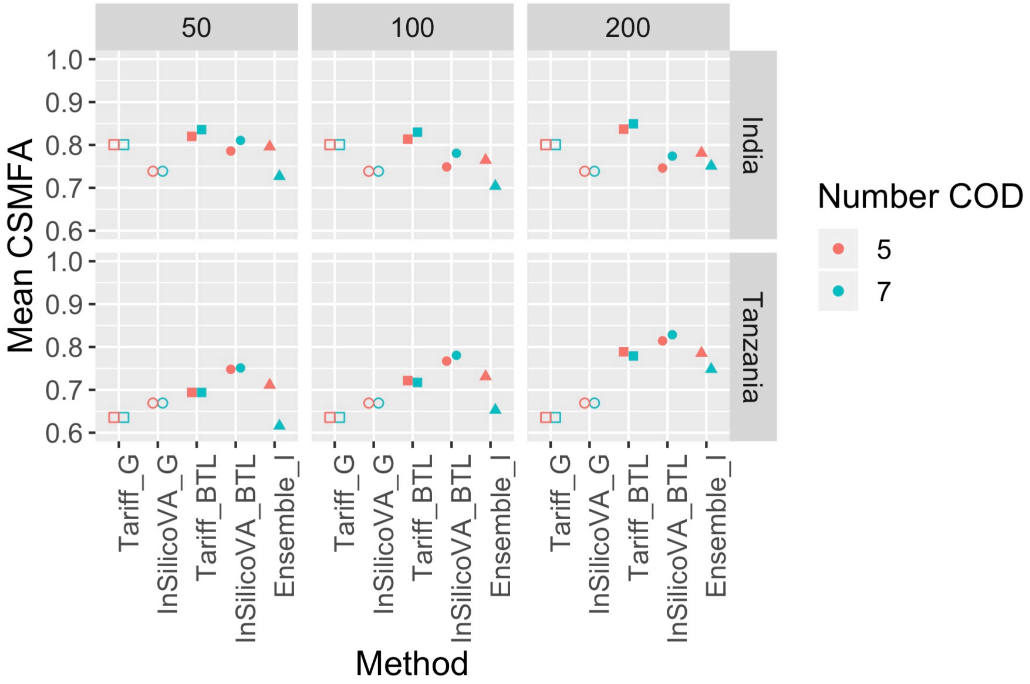

Fig. S9. Comparison of BTL performance with 7 versus 5 cause categories

and 100 when we add additional causes. This is also most likely due to the fact that the ensemble method requires estimating substantially more parameters with an increased number of causes, as compared to the individual algorithm transfer learning. As the sample size of $\mathcal{L}$ grows larger, we are able to better estimate this increased number of parameters.

Additional figures

References

McCormick, Tyler H, Li, Zehang Richard, Calvert, Clara, Crampin, Amelia C, Kahn, Kathleen and Clark, Samuel J. (2016). Probabilistic cause-of-death assignment using verbal autopsies. *Journal of the American Statistical Association* **111**(515), 1036–1049.

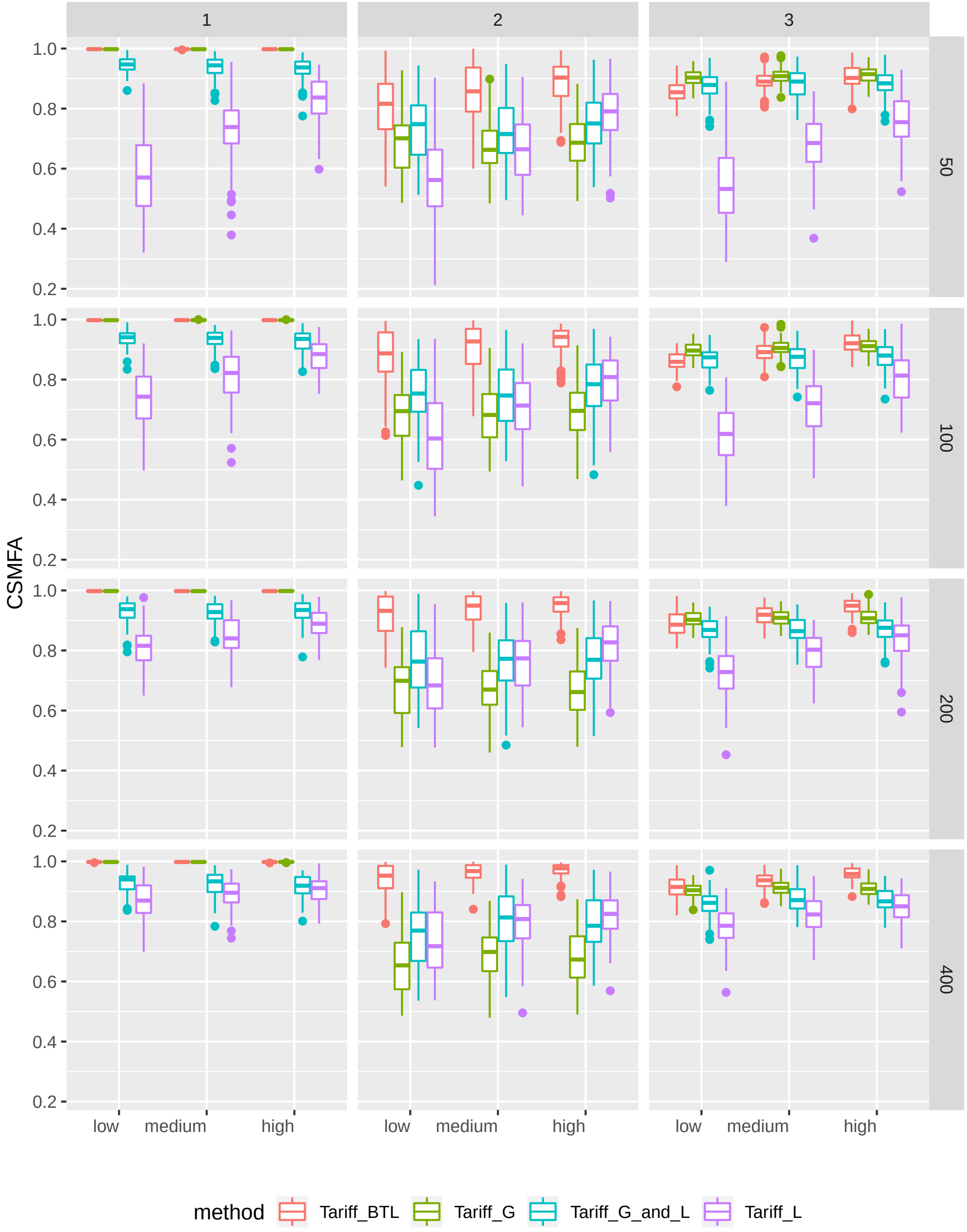

Fig. S10. CSMF for the four Tariff-based methods for data generated using Tariff

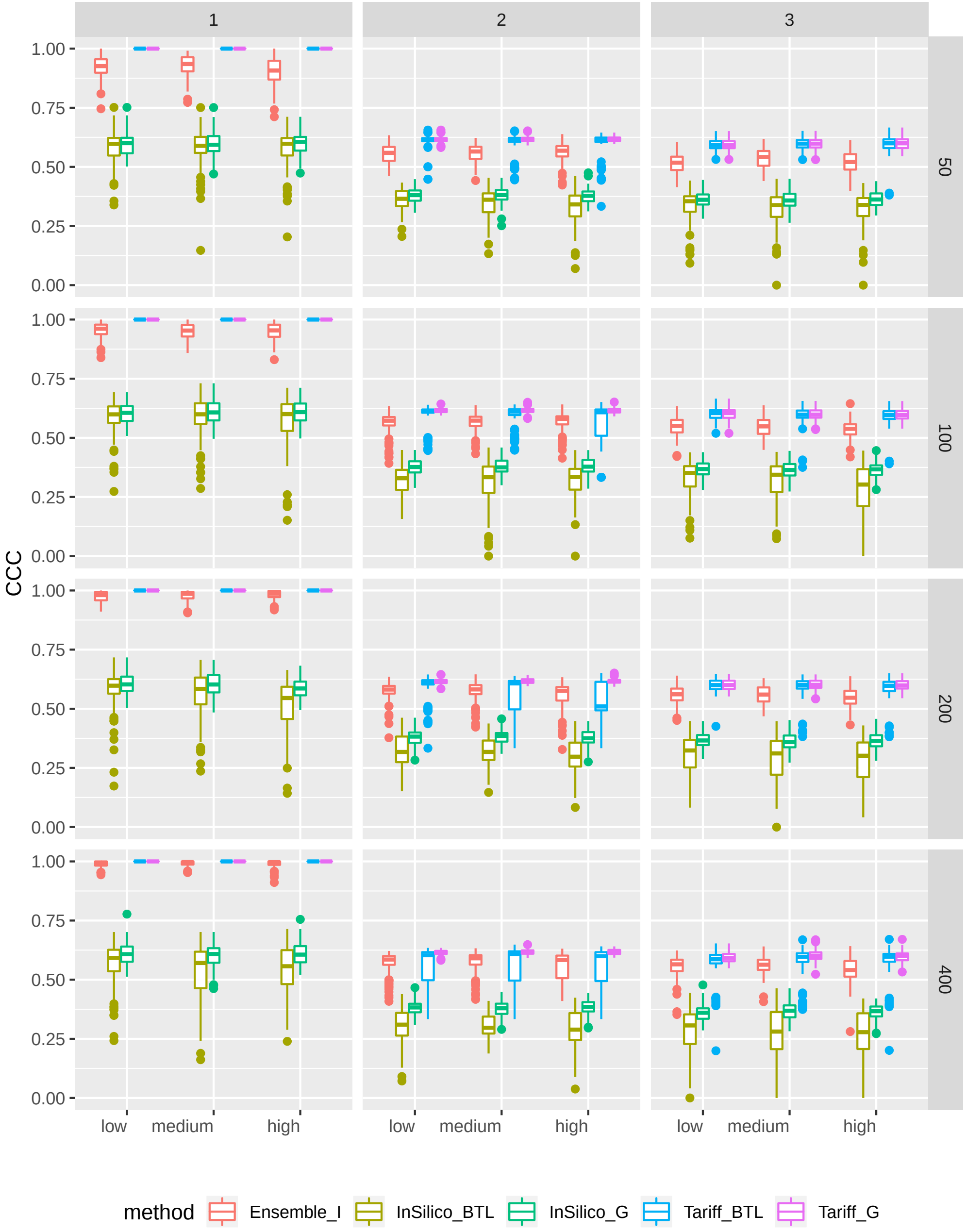

Fig. S11. CCC when data is generated using Tariff

Murray, Christopher JL, Lozano, Rafael, Flaxman, Abraham D, Vahdatpour, Alireza and Lopez, Alan D. (2011). Robust metrics for assessing the performance of different verbal autopsy cause assignment methods in validation studies. *Population health metrics* **9**(1), 28.
